# Combination of gallium citrate and levofloxacin induces a distinct metabolome profile and enhances growth inhibition of multidrug-resistant *Mycobacterium tuberculosis* compared to linezolid

**DOI:** 10.3389/fmicb.2024.1474071

**Published:** 2024-11-29

**Authors:** Oleksandr Ilchenko, Elena Nikolaevskaya, Oksana Zinchenko, Volodymyr Ivanytsia, Cristina Prat-Aymerich, Madeleine Ramstedt, Olena Rzhepishevska

**Affiliations:** ^1^Department of Chemistry, Umeå, University, Umeå, Sweden; ^2^Department of Microbiology, Virology and Biotechnology, Faculty of Biology, Odesa I.I. Mechnikov National University, Odesa, Ukraine; ^3^Odesa Center for Socially Significant Diseases of Odesa Regional Council, Odesa, Ukraine; ^4^CIBER Enfermedades Respiratorias, University Hospital Germans Trias I Pujol, Badalona, Spain; ^5^ECRAID, European Clinical Research Alliance on Infectious Diseases. Julius Center for Health Sciences and Primary Care, University Medical Centre Utrecht, Utrecht University, Utrecht, Netherlands; ^6^Department of Clinical Microbiology, Umeå University, Umeå, Sweden

**Keywords:** levofloxacin, metabolome, drug resistance, central metabolism, *Mycobacterium tuberculosis*, gallium, linezolid, drug–drug interaction

## Abstract

**Introduction:**

Tuberculosis (TB) treatment typically involves a tailored combination of four antibiotics based on the drug resistance profile of the infecting strain. The increasing drug resistance of *Mycobacterium tuberculosis* (*Mtb*) requires the development of novel antibiotics to ensure effective treatment regimens. Gallium (Ga) is being explored as a repurposed drug against TB due to its ability to inhibit *Mtb* growth and disrupt iron metabolism. Given the potential interactions between Ga and established antibiotics, we investigated how a combination of Ga with levofloxacin (Lfx) or linezolid (Lzd) affects the growth and metabolome of a multidrug-resistant (MDR) *Mtb* clinical strain.

**Methods:**

*Mtb* was cultured using a BACTEC 960 system with concentrations of Ga ranging from 125 to 1,000 μM and with 250 to 500 μM of Ga combined with 0.125 mg/L of Lfx or Lzd. For metabolome analysis, the antibacterials were used at concentrations that inhibited the growth of bacteria without causing cell death. Metabolites were extracted from *Mtb* cells and analyzed using chromatography-mass spectrometry.

**Results:**

The MDR *Mtb* strain exhibited a dose-dependent response to Ga. Notably, the enhancement in growth inhibition was statistically significant for the Ga/Lfx combination compared to Ga alone, while no such significance was observed for Ga/Lzd. Moreover, exposure to Ga/Lfx or Ga/Lzd resulted in distinct metabolite profiles. Ga treatment increased the level of aconitate, fumarate, and glucose in the cells, suggesting the inhibition of iron-dependent aconitase and fumarate hydratase, as well as disruption of the pentose phosphate pathway. The levels of glucose, succinic acid, citric acid, and hexadecanoic acid followed a similar pattern in cells exposed to Ga and Ga/Lfx at 500 μM Ga but exhibited different trends at 250 μM Ga.

**Discussion:**

In the presence of Lfx, the *Mtb* metabolome changes induced by Ga are more pronounced compared to those observed with Lzd. Lfx affects nucleic acids and transcription, which may enhance Ga-dependent growth inhibition by preventing the metabolic redirection that bacteria typically use to bypass iron-dependent enzymes.

## Introduction

Tuberculosis (TB) is a bacterial disease that claims the lives of approximately 1.5 million people each year (World Health Organization, 2023). *Mycobacterium tuberculosis* (*Mtb*), the causative agent of TB, can accumulate mutations in its chromosome that lead to drug resistance, resulting in drug-resistant TB (DR-TB). This presents a significant barrier to effective TB control (World Health Organization, 2023; Merker et al., 2020). Treatment for DR-TB typically involves a combination of several antibiotics, usually four, depending on the specific drug resistance pattern (Vanino et al., 2023). New antibiotics are continuously needed to ensure that patients receive an effective and safe treatment regimen.

Gallium (Ga), a chemical element and an iron mimetic, is already used in medical applications. Injectable Ga buffered with citrate is an FDA-approved drug used in scintigraphy (imaging) to assess the location of cancer, nonspecific inflammation, and infectious diseases (Waxman et al., 1984; Liu et al., 2007). Ga nitrate solution (Ganite) is approved for normalizing elevated plasma calcium levels in cancer patients (Patel et al., 2020), containing the following components: Ga nitrate (25 mg), sodium citrate (28.75 mg), and pH-regulating compounds. A clinical trial involving patients with advanced malignant lymphoma concluded that Ga nitrate is well tolerated when administered for a period of 7 days at 300 mg/m^2^/day (Warrell et al., 1983). Additionally, a pharmacokinetic and safety study was conducted on cystic fibrosis patients using 200 mg/m^2^/day of Ga nitrate for 5 days (Goss, 2022).

Currently, Ga is being investigated as a repurposed drug for treating bacterial infections, including tuberculosis (Bernstein, 1998; Bonchi et al., 2014) and infections caused by non-tuberculous mycobacteria (CTV, 2024). Ga can inhibit growth and kill *Mtb* in liquid culture (Machado et al., 2014), macrophages, and animal models (Olakanmi et al., 2013; Olakanmi et al., 2000). Ga can halt the activity of iron-containing enzymes by substituting iron and cause metabolic changes in bacteria, including *Mtb* (Bernstein, 1998; Olakanmi et al., 2013; Rzhepishevska et al., 2011). Since TB is treated by several drugs simultaneously, drug–drug interaction is an important issue (Riccardi et al., 2020). Additionally, established antibiotics can also cause metabolic changes in bacteria (Belenky et al., 2015; Knoll et al., 2021). Hence, a combination of Ga with established antibiotics might lead to enhanced or decreased activity due to metabolic interactions or other reasons. For example, synergistic effects were reported against biofilms of *Pseudomonas aeruginosa* as well as on *Mtb* growth (Rzhepishevska et al., 2014; Leitao et al., 2023). However, a potential interaction, including the metabolic interplay between Ga and established anti-TB medicines, has not been studied.

Mechanisms of drug activity and the traits of bacterial physiology during exposure to antibacterial agents can be successfully elucidated by metabolomics and targeted analysis of metabolites (Rinschen et al., 2019; Kontsevaya et al., 2021; Reithuber et al., 2020; Patil and Jain, 2021). In this study, we used metabolomics together with drug susceptibility testing to better understand the effect of Ga on multidrug-resistant (MDR) *Mtb* in combinations with 10% minimal inhibitory concentrations of levofloxacin (Lfx) and linezolid (Lzd), which both are well-established drugs for the treatment of MDR-TB.

## Materials and methods

### Strains and growth conditions

A multidrug-resistant (MDR) *Mtb* strain 6,284 was used for growth inhibition testing. The strain was isolated at the Odesa Center for Socially Significant Diseases, Odesa, Ukraine, with genome sequencing performed as outlined in the following section. *Mtb* strain 6,284 was also used for metabolome analysis. However, to increase the number of samples for Ga 500 μM, six additional extracts of another MDR *Mtb* clinical strain 5,657 cultured in the presence of Ga 500 μM were used. Bacteria were grown at 37°C using the BD BBL™ BACTEC 960 system with MGIT™ tubes (Mycobacteria Growth Indicator Tube 7 mL, BD BBL™) complemented with the BD BACTEC™ MGIT™ 960 Supplement kit, in triplicate. PANTA™ antibiotic mixtures were prepared by reconstituting a lyophilized vial of BBL MGIT PANTA™ that contained a lyophilized mixture of antimicrobial agents [Polymyxin B (6,000 units), Amphotericin B (20 g), Nalidixic acid (1.1 g), Trimethoprim (0.03 g), Azlocilin (0.1 g)] with 15 mL of BACTEC™ MGIT™ Growth Supplement that contained 15 mL Middlebrook OADC enrichment [Bovine albumin (50 g), dextrose (20 g), poloxyethylene stearate (POES) (1.1 g), catalase (0.03 g), oleic acid (0.1 g)].

To determine the minimal inhibitory concentration for Ga, the MGIT™ media was complemented with Ga at concentrations ranging from 125 μM to 1,000 μM. pH was measured in MGIT™ tubes at different concentrations of Ga (125–1,000 μM) with Metler Toledo SevenEasy pH, Switzerland.

During testing for minimal inhibitory concentrations, MGIT™ medium contained Ga(NO_3_)_3_ (125 to 1,000 uM) and citrate (0.625 to 5 mM). Citrate was added to citrate controls at 0.625 to 5 mM. Citric acid increased the acidity of the medium. We chose to conduct experiments at an acidic pH because of two reasons. Firstly, using low pH provides a more stable concentration of Ga in the growth medium and better availability for bacteria (Rzhepishevska et al., 2011). Secondly, acidic pH is relevant for antibacterial testing since the macrophage environment where *Mtb* resides as an intracellular pathogen is acidic (Gouzy et al., 2021; Early et al., 2019). To account for the possible effect of pH and citrate on *Mtb* cells, we used citrate-containing MGIT™ medium as a control.

**Figure 1:**
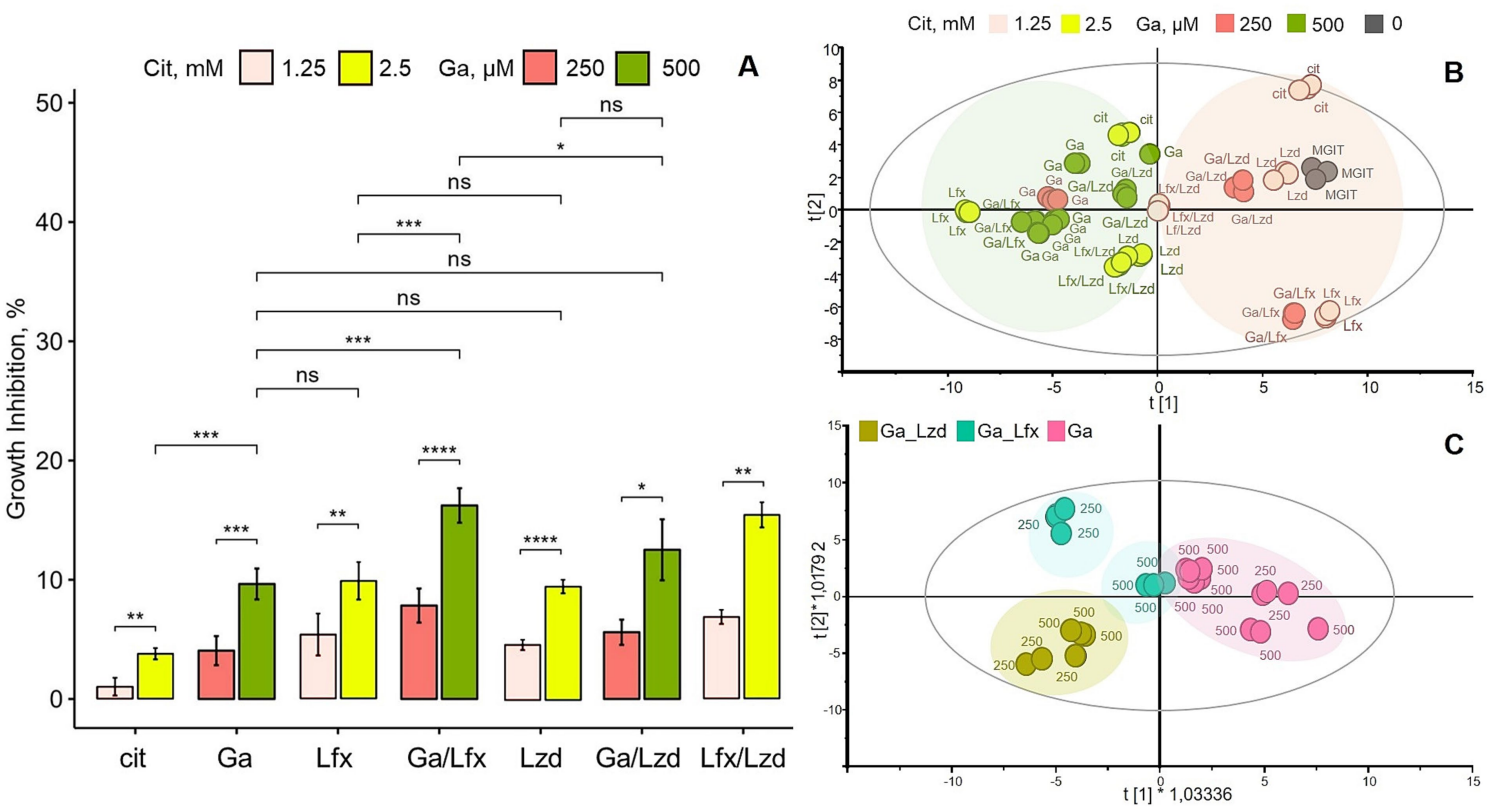
Influence of Ga and its combination with Lfx or Lzd on *Mtb* growth and metabolome; **(A)** growth inhibition of *Mtb* MDR strain in MGIT™ medium with Ga, Lfx or Lzd, presented as percent inhibition of growth in standard MGIT™ medium; experimental conditions labeled as follows: MGIT™ medium with citrate (cit); Ga citrate (Ga); Lfx with citrate (Lfx), Ga citrate with Lfx (Ga/Lfx); Ga citrate with Lzd (Ga/Lzd), Lzd with citrate (Lzd); Lfx with Lzd and citrate (Lfx/Lzd). Ga was used at 250 and 500 μM. Citrate was used at 1.25 and 2.5 mM (as controls to Ga 250 and 500 μM), while Lfx or/and Lzd were used at 0,125 mg/L. Statistical significance after false discovery rate correction: ns >0.05, * *p* ≤ 0.05, ** *p* ≤ 0.01, ***p ≤ 0.001, ****p ≤ 0.0001; **(B)** scores plot of an unsupervised PCA model summarizing all GC–MS metabolomics data; R2X [1] = 0.482; R2X [2] = 0.21; Ellipse: Hotelling’s T2 (95%); the model shows that samples (metabolomes of *Mtb* grown at different experimental conditions) cluster according to two concentrations of Ga and/or citrate; Ga was used at 250 and 500 μM, citrate was used at 1.25 and 2.5 mM (as controls to Ga 250 and 500 μM), while Lfx or/and Lzd were used at 0,125 mg/L; the experimental conditions are labeled as in **A** with the addition of standard MGIT™ medium (MGIT); color code of **B** corresponds to **A**; the loadings plot of this model showing metabolites responsible for clustering is provided as a Supplementary Figure 2; **(C)** scores plot of a supervised OPLS-DA model built with GC–MS metabolomics data; the model illustrates differences between three experimental conditions at two concentrations of Ga: Ga (Ga); Ga with Lfx (Ga/Lfx); Ga with Lzd (Ga/Lzd); the model is scaled proportionally to R2X; R2X [1] = 0,289; R2X [2] = 0,241; Ellipse: Hotelling’s T2 (95%); CV-ANOVA *p* = 0.0001; the model identifies two concentration-dependent subclusters in samples with Ga/Lfx: pink – gallium 250 and 500 μM; green-blue – Ga/Lfx 250 and 500 μM; green-yellow – Ga/Lzd 250 and 500 μM; the loadings plot of this model showing metabolites responsible for clustering is provided as a Supplementary Figure 4.

We set up a series of preliminary growth inhibition experiments to choose the concentrations of Ga, Lzd, and Lfx for metabolite analysis. We defined ~ 20% growth inhibition by Ga 500 uM/Lfx as a maximum threshold for inhibition (Figure 1). Even a smaller, 1.5-times increase of Ga concentration beyond 500 uM (Supplementary Figure 1) sharply impacted bacterial growth by almost 50%, and in combination with Lfx, this inhibition would go over 50%. Thus, the choice of Ga, Lzd, and Lfx concentrations used in this study is based on the *Mtb* growth inhibition pattern and the need to meet sample quality and quantity for metabolite analysis. We use the term “moderate inhibition” in this manuscript to refer to the observed concentration-dependent, statistically significant growth inhibition compared to control that is below the defined maximum threshold of inhibition, i.e., below 20%.

For Ga combinations with antibiotics, we used the following experimental conditions: Ga citrate (Ga); Ga with Lzd (termed Ga/Lzd), Ga with Lfx (termed Ga/Lfx); Lzd with citrate (termed Lzd); Lfx with citrate (termed Lfx), Lfx with Lzd and with citrate (termed Lfx/Lzd), MGIT™ media with citrate (termed cit), standard MGIT™ media (termed MGIT). Ga was used at 250 and 500 μM. Citrate was used at 1.25 and 2.5 mM (as controls to Ga250 and 500 μM), while Lfx or/and Lzd were used at 0.125 mg/L. Hence, each of the experimental conditions, apart from the one termed “MGIT,” was performed at two concentrations of Ga (250 and 500 μM) or two concentrations of citrate (1.25 and 2.5 mM) if this experimental condition did not include Ga. Each MGIT™ tube was inoculated with 0.1 mL of *Mtb* strain suspension with OD = 0.1 ± 0.05.

### Whole genome sequencing and drug resistance mapping

Whole genome sequencing (WGS) of strain 8,284 was done using NextSeq 500 and Nextera XT library preparation kit. The mutations conferring drug resistance were mapped as previously described (Merker et al., 2020).

### Growth inhibition assay

The growth inhibition in percent of the growth of *Mtb* strain in a standard MGIT™ media was calculated by using Time to Detection (TTD) values from the BACTEC™ 960 instrument modified from Diacon et al. (2010). The BACTEC™ MGIT™ 960 incubator is an automated instrument that uses an oxygen sensor and fluorescence to monitor the growth of mycobacteria in culture. The instrument reports TTD measured in hours when the growth units reach the growth detection threshold. In our experiment, if the growth units for a sample did not reach the threshold within 1023 h (42 days), the sample was considered negative for growth according to the protocol used in the clinical testing.

When testing compounds for growth inhibition in the BACTEC™ MGIT™ 960 system, we considered the absence of growth after 1023 h in an inoculated tube containing a specific compound (e.g., Ga) to have shown 100% growth inhibition. A positive control was included to account for the standard growth rate of the *Mtb* strain we used. This positive control was a tube with MGIT™ growth media without inhibitory compounds inoculated with the same number of bacteria as the tubes with a test compound. The average TTD for a positive control (105.5 h) was subtracted from the TTD for the “no growth” control (1023 h). The value of 1023 h was constant for all tests using the BACTEC™ 960 system. However, the TTD for a positive control varied depending on the strain of *Mtb* and the number of cells inoculated (OD of inoculum). Hence, it should be noted that for this study, 100% growth inhibition corresponded to no observed growth after 917.5 h. To calculate the percentage of growth inhibition for every condition, the positive control TTD was subtracted from TTD for every experimental tube and divided by 100% of growth inhibition in hours using the following equation:


$$GI=\frac{\left(TTD_{\text{exp}}-TTD_{ctr}\right)}{\frac{TTD_{\text{max}}-TTD_{\mathrm{positive}\_\min}}{100}}$$

*GI*, Growth Inhibition %; *TTD_exp_*, TTD (experimental tube); *TTD_ctr_*, TTD (positive control); *TTD_positive_min_* – 105.5 h; *TTD_max_* – 1023 h.

### Gas chromatography–mass spectrometry

We analyzed a broad range of metabolites from *Mtb* cells using gas chromatography–mass spectrometry (GC–MS) as previously described (Rzhepishevska et al., 2011) with minor modifications. Sample preparation included cell harvesting, sample extraction, and sample derivatization. For biosafety purposes, sample extraction was done at the clinical laboratory, a BSL3-certified facility where bacteria were cultured. *Mtb* cells were harvested by centrifugation from three independent culture tubes. After harvesting, cells were washed twice with 2 mL ice-cold NaCl 0.9% and extracted with 450 μL of extraction mixture [MeOH/H_2_O (90:10 v/v (Fisher Chemicals))] including internal standards (7 ng/ul). We added a tungsten bead (Qiagen) to each tube and vortexed the tubes for 2 min to break the cells and facilitate the release of the metabolites. Further, samples were incubated on ice for 2 h and centrifuged for 10 min, at 4°C, at 14000 rpm. The supernatant containing extracted metabolites was transferred to a fresh tube and kept at −20 or − 80°*C. prior* to derivatization, 100 μL of the supernatant was transferred to GC-vials (Scantec Nordic/Thermo Scientific). The samples were evaporated for 2 h using the miVac Quatro Concentrator (Gene Vac) until dryness. For derivatization, methoxamine (15 μL) was added to the vial with a dried sample and shaken for 10 min using Vortex mixer multi (VWR) and incubated in an oven at 70°C for 1 h in an oven (Memmert GmbH/Fisher Scientific). The samples were incubated at room temperature for 16 h. Further, 30 μL of a 50:50 mix of N-Methyltrimethylsilyltrifluoroacetamide (MSTFA) 1% and heptane (including methyl stearate 15 ng/ml) was added to each sample, vortexed and incubated at room temperature for 1 h before the analysis. For alkane series standards, a mix of C8-C20 (10 μL), C21-40 (10 μL), and heptane (30 μL), all purchased from Sigma-Aldrich/Merck, was used. GC–MS: the samples were analyzed using Pegasus® BT GC-TOFMS, Benchtop Gas Chromatography Time-of-Flight Mass Spectrometer. We used a fused silica capillary column (10 m × 0.18 mm × 0.18 μm i.d.) with a stationary phase DB5-MS (0.18 μm) and helium as a mobile phase. An aliquot of 0.5 μL of each sample was collected using an L-PAL3 Leco autosampler and injected into an Agilent 7809B gas chromatograph coupled to a mass analyzer. QC samples (quality control) were prepared by pooling 50 μL of extracted samples.

The raw files were exported from the ChromaTOF software in NetCDF format to MATLAB R2016a (Mathworks, Natick, MA, USA) for data compression, base-line correction, chromatogram alignment, and annotation of the mass spectra by comparisons of their retention index and mass spectra to the in-house libraries. Further, the list of resulting metabolites was imported into NIST MS 2.2 software to validate the annotation based on match score and reverse match score. Peak areas of annotated metabolites were normalized between all experimental conditions by the following formula:


$$\mathrm{Np}a_{i}=\frac{pa_{i}}{\sum_{i=1}^{n}\left(pa_{i}\right)/k}$$

$\mathrm{Np}a_{i}$ , normalized peak area; $pa_{i}$ , non-normalized peak area; *k*, coefficient is equal to 1*10^5^.

### Liquid chromatography-mass spectrometry

For liquid chromatography-mass spectrometry (LC–MS), we followed the same procedure as for GC–MS when it came to the cultivation of bacteria and metabolite extraction (see section above). Briefly, *Mtb* cells were harvested by centrifugation from three independent culture tubes. After harvesting, cells were washed twice with 2 mL ice-cold NaCl 0.9% and extracted with 450 μL of extraction mixture [MeOH/H2O (90:10 v/v (Fisher Chemicals))]. From this point, the protocol for LC–MS sample preparation differs from the GC–MS protocol. A 30 μL aliquot of an extract from each experimental condition was pooled for quality control (QC). Further, 200 μL of the extract from each experimental condition and QCs were transferred to chromatographic vials and evaporated to dryness in a speed-vac concentrator. Each sample was re-suspended in 10 + 10 μL methanol and water and analyzed using a 1,290 Infinity liquid chromatograph coupled to a Q-TOF 6546 mass spectrometer (Agilent) in a positive mode. The chromatographic separation was performed on an Agilent 1,290 Infinity UHPLC-system (Agilent Technologies, Waldbronn, Germany). 2 μL of each sample were injected onto an Acquity UPLC HSS T3, 2.1 × 50 mm, 1.8 μm C18 column in combination with a 2.1 mm x 5 mm, 1.8 μm VanGuard precolumn (Waters Corporation, Milford, MA, USA) held at 40°C. The gradient elution buffers were A (H2O, 0.1% formic acid) and B (75/25 acetonitrile: 2-propanol, 0.1% formic acid), and the flow rate was 0.5 mL min^−1^. The compounds were eluted with a linear gradient consisting of 0.1–10% B over 2 min, B was increased to 99% over 5 min and held at 99% for 2 min; B was decreased to 0.1% for 0.3 min, and the flow rate was increased to 0.8 mL min^−1^ for 0.5 min; these conditions were held for 0.9 min, after which the flow rate was reduced to 0.5 mL min^−1^ for 0.1 min before the next injection.

The compounds were detected using an Agilent 6,546 Q-TOF mass spectrometer equipped with a jet stream electrospray ion source. For accurate mass measurements, a reference interface was connected, and reference ions, purine (4 μM) and HP-0921 (Hexakis (1H, 1H, 3H-tetrafluoropropoxy) phosphazine) (1 μM), were directly introduced into the mass spectrometer at a flow rate of 0.05 mL min^−1^ for internal calibration. The purine ions monitored were m/z 121.05 and m/z 119.03632.

The gas temperature was maintained at 150°C, with a drying gas flow of 8 L min^−1^ and a nebulizer pressure of 35 psig. The sheath gas temperature was set to 350°C, with a sheath gas flow of 11 L min^−1^. The capillary voltage was maintained at 4000 V, while the nozzle voltage was set at 300 V. The fragmentor voltage was 120 V, the skimmer voltage was 65 V, and the OCT 1 RF Vpp was 750 V.

The collision energy was fixed at 0 V. The m/z range was set from 70 to 1700, and data were collected in centroid mode with an acquisition rate of 4 scans s^−1^ (1977 transients/spectrum). MSMS analysis was conducted on QC samples for identification purposes.

All LC–MS data processing was done both in a target and an untargeted mode using the Agilent Masshunter Profinder version B.10.00 (Agilent Technologies Inc., Santa Clara, CA, USA). For target processing, an in-house library of metabolites commonly found in biological samples and a library of standards run on the same system with the same chromatographic and mass-spec settings were used. The metabolites were identified based on MS, MSMS, and retention time information. All data files were processed using the Batch Recursive Feature Extraction algorithm within Masshunter Profinder for the untargeted data. All unknown extracted molecular features in Agilent Profinder were annotated in SIRIUS software (Lehrstuhl Bioinformatik, Jena).

### Data analysis

To follow metabolite patterns derived by GC/LC–MS at different conditions, we applied univariate analysis, principal component analysis (PCA), orthogonal projection to latent structures discriminant analysis (OPLS-DA), and modeling in SIMCA 17 (Sartorius). Volcano plots, bar plots, and jitter plots were visualized with the ggplot2 package in R. For the GC–MS data, volcano plots were built separately for the concentrations of 250 and 500 μM Ga. In contrast, the LC–MS data acquired from cells grown with 250 and 500 μM Ga were pooled together. The fold change values and statistical significance were determined using ANOVA and analyzed through the “Mevis” R script (Ilchenko, 2021).

## Results

### Drug resistance profile of the *Mtb* strain used in the growth inhibition assay

In *Mtb*, drug resistance mutations are located on the chromosome. Using whole genome sequencing, the clinical *Mtb* strain 6,284 was classified as Beijing genotype (lineage 2.2.1) multidrug-resistant (MDR) based on the following detected mutations conferring resistance to a range of antibiotics: *fabG1* -15c > t and *katG* S315T (isoniazid); *rpoB* S450L (rifampicin); *rpsL* K88R (streptomycin); *embA* -12c > t (ethambutol); *pncA* L151 (pyrazinamide). We also confirmed that this strain, phenotypically sensitive to levofloxacin (Lfx) and linezolid (Lzd), did not have any mutations known to be associated with Lfx or Lzd resistances. This means that isoniazid, rifampicin, streptomycin, ethambutol, and pyrazinamide cannot inhibit the growth of this strain and cannot be used to treat TB caused by this strain. Lfx and Lzd can be used for treatment and growth inhibition assays.

### Minimal inhibitory concentration of Ga in MGIT™ media

The minimal inhibitory concentration (MIC) of Ga often depends on the composition of the growth medium, including the concentration of iron (Rzhepishevska et al., 2011). We tested the response of *Mtb* to Ga in MGIT™ medium that is used routinely in clinical work to ensure good Mtb growth and a high iron content. The *Mtb* strain 6,284 responded to Ga citrate (125–1,000 μM) dose-dependently (Supplementary Figure 1A). Respective concentrations of citrate in control tubes were slightly inhibiting. However, even the highest concentration of citrate used (5 mM) inhibited the *Mtb* growth by only 50% (Supplementary Figure 1A). Ga at 1000 μM completely prevented the *Mtb* growth in the MGIT™ medium, while Ga at 750 μM inhibited the growth by roughly 45%. Ga at concentrations 125, 250, and 500 μM showed moderate growth inhibition (Supplementary Figure 1A). Hence, here, Ga 1,000 μM is the MIC at the conditions used in this study.

Additionally, Ga citrate increased the acidity of the growth medium. The standard MGIT™ medium had a pH of 6.7, while the MGIT™ medium with 500 and 1,000 μM of Ga citrate had a pH of 5.7 and 4.2, respectively (Supplementary Figure 1B). Based on the obtained growth inhibition range (Supplementary Figure 1A), the concentrations 250 and 500 μM were chosen for further testing of the Ga growth inhibition in combination with antibiotics and for metabolomics analyses. These concentrations were chosen to inhibit the growth substantially but still keep bacteria alive and replicating to allow monitoring of the effect of Ga and antibiotics on *Mtb* metabolism.

### A combination of Ga with Lfx results in stronger growth inhibition of *Mtb*

Subsequently, we investigated the growth inhibition of Ga in combination with established antibiotics used routinely to treat MDR-TB. Importantly, Ga, Lfx, and Lzd, used on their own, resulted in a significantly increased growth inhibition compared to the control (MGIT ™ with a citrate buffer) but did not differ in their ability to inhibit the growth of *Mtb* at given concentrations (Figure 1A). In the presence of fixed concentrations of Lzd or Lfx (0.125 mg/L), the MDR-TB strain responded to Ga treatment in a dose-dependent manner, meaning that the growth inhibition by Ga 500 μM was stronger than by Ga 250 μM (Figure 1A). In the presence of Ga/Lzd, the level of growth inhibition was similar to that of Ga or Lzd alone (Figure 1A). On the other hand, the Ga/Lfx combination resulted in stronger growth inhibition. At 500 μM, the combination of Ga with Lfx resulted in significantly increased growth inhibition compared to Ga alone or Lfx alone (Figure 1A). One sample was excluded from the data set presented in Figure 1A because it showed no growth and was identified as an outlier by the Grubbs’ test. The combination of Ga with Lfx, thus, had a stronger growth inhibition effect on the *Mtb* strain than Ga or Lfx alone at the same concentrations or the combination of Ga with Lzd.

### The metabolite profile of *Mtb* cells grown in the presence of Ga is concentration dependent

To assess the metabolic response of *Mtb* cells grown in the presence of two concentrations of Ga and combinations of Ga with Lfx and Lzd (Ga/Lfx and Ga/Lzd), we constructed a PCA model that included metabolite data (peak areas corresponding to relative concentrations) (Supplementary Table 1) of all 60 metabolites measured by GC–MS for all of these experimental conditions (Figure 1B; Supplementary Figure 2; Supplementary Table 1). The score plot of the model shows that the samples are separate in the first component of the model, likely based on the concentration of Ga citrate and citrate (Figure 1B). Hence, the concentration of Ga and citrate affects the pattern of *Mtb* metabolites more strongly than the presence of Lzd and Lfx. This pattern thus may correspond to the dose-dependent effect of Ga, citrate, and/or pH observed in the growth inhibition assays (Figure 1A). The loading plot of the PCA model (Supplementary Figure 2) shows that a larger diversity of metabolites is associated with the 250 μM Ga compared to 500 μM. The larger diversity of metabolites at 250 μM Ga is not surprising as the inhibitory effect is lower. Thus, the bacteria may use a wider range of metabolic pathways compared to the growth at a higher concentration of Ga. However, the levels of glucose, mannose, galactose, citric acid, aconitic acid, alanine, and a few other metabolites were increased at 500 μM Ga, suggesting that a higher concentration of Ga can influence certain metabolic pathways (Supplementary Figure 2).

Further, we assessed whether the presence of citrate and the difference in pH could overlap with the Ga effect on the *Mtb* metabolome. For this, we constructed a separate OPLS-DA model where we compared metabolomics data from cells grown in the presence of citrate only (1.25 and 2.5 mM) with those grown in the presence of Ga citrate (250 and 500 μM) (Supplementary Figures 3A,B). The model showed that Ga was associated with aconitate, glucose, mannose, galactose, alanine, citric, and fumaric acids. On the other hand, arabinose was associated with the presence of citrate and may potentially be a product of citrate metabolism. Asparagine and the unknown metabolites UPSC_20067 and UPSC_10164 were associated with citrate and Ga at higher concentrations (2.5 mM and 500 μM, respectively). They may be part of the cell response to increased acidity (Supplementary Figures 3A,B).

Subsequently, we applied a supervised OPLS-DA analysis (Figure 1C; Supplementary Figure 4) to assess whether we could detect distinct metabolite patterns for Ga and/or its combinations with Lfx or Lzd at both concentrations of Ga tested (250 and 500 μM). The score plot of the model (Figure 1C) shows that samples cultured in the presence of Ga alone and Ga/Lzd form distinct clusters. However, the samples cultured in the presence of Ga/Lfx split into two smaller clusters based on Ga concentration (Figure 1C). This indicates that for the cells grown in the presence of Ga/Lfx, the metabolite pattern may be influenced in different ways depending on the concentration of Ga or pH.

### Cells grown with Ga alone and Ga/Lfx display a similar metabolite pattern at 500 μM

**Figure 2:**
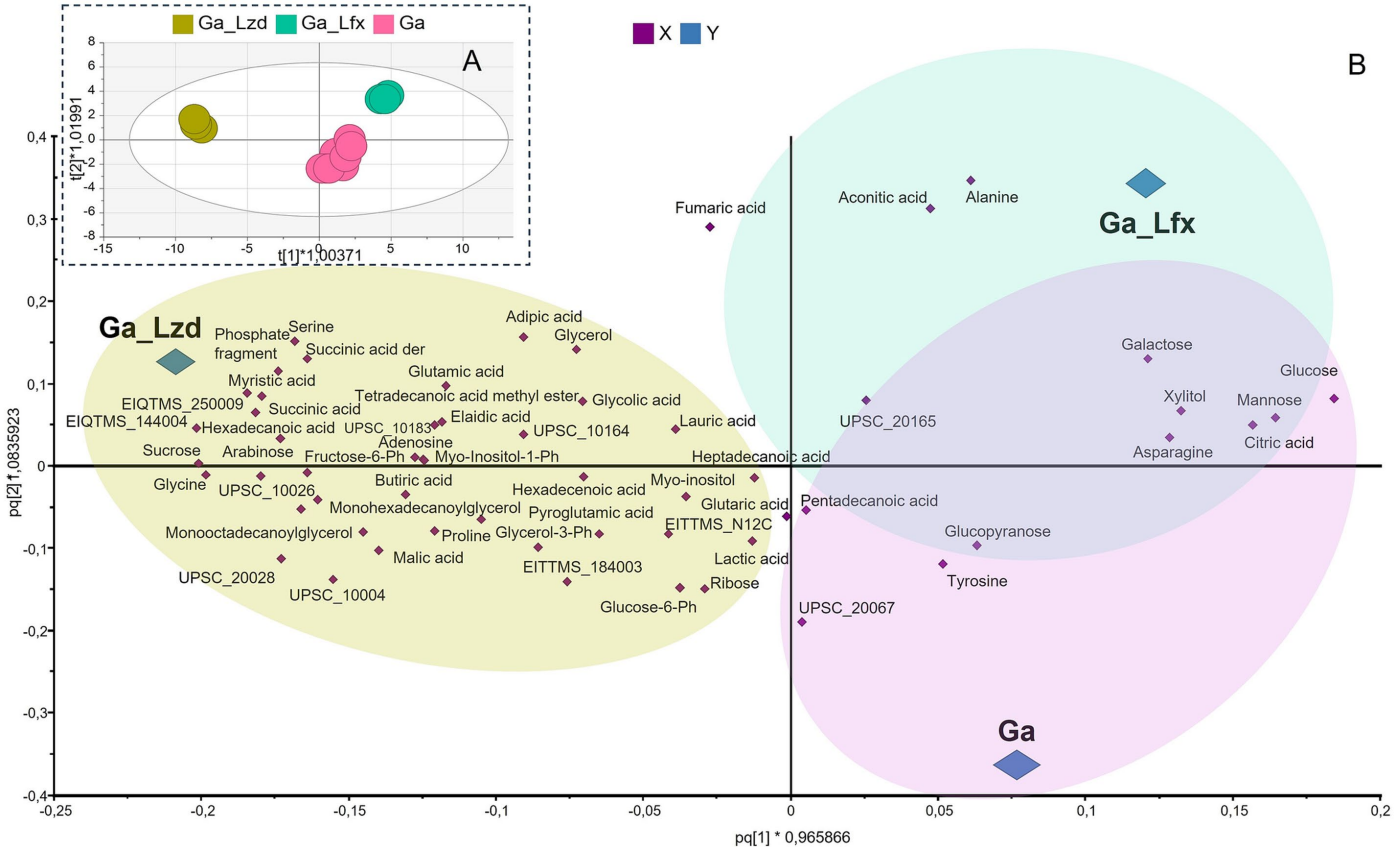
Influence of Ga 500 μM and its combination with Lfx or Lzd on the metabolome of *Mtb*; **(A)** scores plot of a supervised OPLS-DA model, scaled proportionally to R2X; R2X [1] = 0.347; R2X [2] = 0.0845; Ellipse: Hotelling’s T2 (95%); CV-ANOVA *p* = 0,001; the model shows the separation of samples (*Mtb* metabolomes) based on experimental conditions: pink – Ga 500 μM; green-blue – Ga/Lfx 500 μM; green-yellow – Ga/Lzd 500 μM; the model identifies Ga/Lzd as a group distinct from Ga/Lfx and Ga alone; Ga/Lfx and Ga alone show overlapping metabolite profile; **(B)** the loadings plot of this model showing metabolites responsible for clustering; colored areas on the plot are used for visualization and matching the scores plot groups to the loadings plot.

Further focusing on the concentration effect, in a separate OPLS-DA model, we analyzed the samples where we saw the strongest growth inhibition, i.e., metabolomes of *Mtb* cells grown at 500 μM Ga (with and without Lfx or Lzd) (Figures 2A,B). Here, the metabolomes of cells grown in the presence of Ga/Lzd separate from those grown in the presence of Ga and Ga/Lfx in the first component of the model (Figure 2A). The model reveals a partially overlapping metabolite pattern for metabolomes of cells grown with Ga/Lfx or with Ga alone. It appears (Figure 2B) that samples grown in the presence of Ga/Lzd are associated with a larger variety of metabolites than those grown in the presence of Ga/Lfx (Figure 2B). In this plot, the levels of glucose, mannose, galactose, citric acid, aconitic acid, alanine, and asparagine were increased in cells cultured in the presence of 500 μM Ga (Figure 2B).

**Figure 3:**
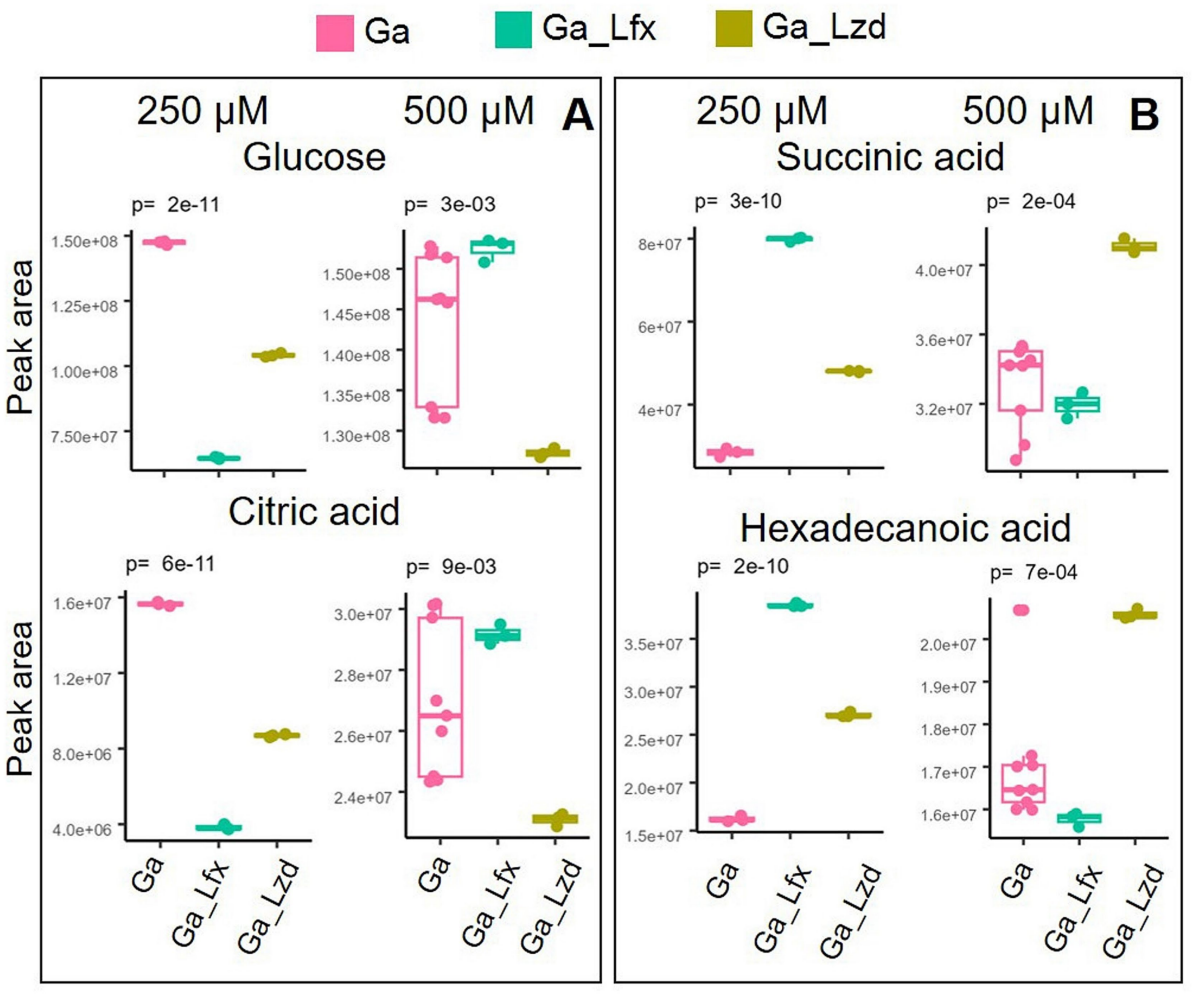
Two patterns of metabolite expression in the presence of Ga/Lfx 250 and 500 μM compared to other experimental conditions; **(A)** metabolites that increase their level in Ga/Lfx 500 μM compared to Ga/Lfx 250 μM; **(B)** metabolites that decrease their level in Ga/Lfx 500 μM compared to Ga/Lfx 250 μM; both in **A** and **B** panel, the peak areas of metabolites measured in cells exposed to Ga/Lfx 500 μM follow the levels of peak areas of metabolites measured in cells exposed to Ga 500 μM.

Normalized peak areas for metabolites altered by exposure to Ga, Ga/Lfx, and Ga/Lzd (derived from the PCA/OPLS-DA models) showed a distinct pattern when cells cultured with Ga 250 and 500 μM were compared (Figure 3). At 250 μM Ga, peak areas of glucose and citrate were lower in cells grown with Ga/Lfx compared to those grown with Ga alone. However, at 500 μM Ga, these peak areas in cells grown with Ga/Lfx increased to similar levels as in cells grown with Ga alone (Figure 3). Peak areas of succinic acid and hexadecenoic acid at 250 μM Ga were higher in cells grown with Ga/Lfx compared to those grown with Ga alone, but, at 500 μM Ga, these peaks decreased to levels similar to those in cells grown with Ga alone (Figure 3). Additionally, alanine, a metabolite increased in cells treated with Ga, exhibited a different pattern in cells grown with Ga/Lfx (Supplementary Figure 5).

**Figure 4:**
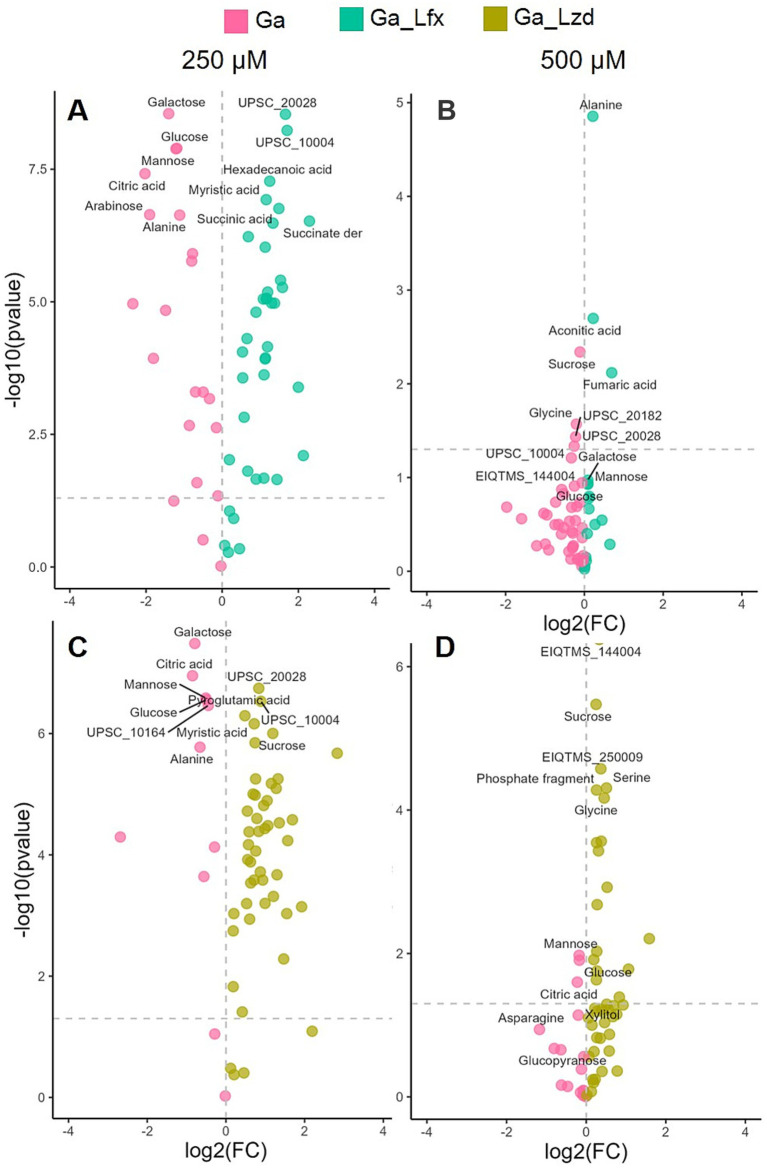
Effect of Ga (250 and 500 μM) and its combination with Lfx or Lzd on the *Mtb* metabolome analyzed by GC–MS Volcano plot analysis identifies the most significant metabolites (marked with annotations) for every growth condition based on their *p*-value and fold change (FC); **(A)** metabolome of cells exposed to Ga compared to Ga/Lfx 250 μM; **(B)** metabolome of cells exposed to Ga compared to Ga/Lfx 500 μM; **(C)** metabolome of cells exposed to Ga compared to Ga/Lzd 250 μM; **(D)** metabolome of cells exposed to Ga compared to Ga/Lzd 500 μM pink – Ga 250 and 500 μM; green-blue – Ga/Lfx 250 and 500 μM; green-yellow – Ga/Lzd 250 and 500 μM; detailed information on all the metabolites used in the volcano plot analysis with FC and *p* values can be found in Supplementary Table 2.

Since in the OPLS-DA model (Figure 2), Ga alone and Ga/Lfx at 500 μM had an overlapping metabolite profile, we compared different growth conditions pairwise using volcano plots (Figure 4; Supplementary Table 2). The analysis identified glucose, galactose, and mannose as metabolites, showing increased levels during Ga exposure at 250 μM compared to exposure to the same Ga concentration combined with Lfx or Lzd (Figure 4; Supplementary Table 2). At 250 μM Ga, the combinations of Ga with Lfx or Lzd resulted in a similar profile of significant metabolites. However, succinate and a succinate derivative were increased in combination with Ga/Lfx but not in Ga/Lzd (Figure 4; Supplementary Table 1). At 500 μM, glucose and mannose were still among the significant metabolites during the exposure to Ga alone compared to Ga/Lzd. The exposure to Ga/Lzd increased levels of amino acids such as glycine and serine, as well as some metabolites that we could not annotate. The exposure to Ga/Lfx at 500 μM showed increased levels of alanine, aconitate, and fumarate (Figure 4; Supplementary Table 2). This indicates increased inhibition of aconitase and fumarate hydratase, which are iron-containing enzymes connected to the tricarboxylic acid cycle (TCA) (Olakanmi et al., 2013). An OPLS-DA model where we compared metabolomics data of cells cultured in the presence of sub-inhibitory concentrations of Lfx with those cultured in the presence of Lzd showed that aconitic acid levels were increased in cells exposed to Lfx (Supplementary Figure 6). Thus, we concluded that Lfx exposure resulted in a similar metabolite profile of the *Mtb* cells as in those exposed to Ga alone. Similarly to OPLS-DA, the diversity of metabolites significantly affected by antibacterials was decreased in the volcano plot analysis at 500 μM Ga compared to 250 μM (Figure 4; Supplementary Table 2), which potentially illustrates the overall inhibiting effect of Ga on cell metabolism. However, for Ga/Lzd exposure, the diversity of metabolites was higher than for Ga/Lfx.

### Additional metabolites associated with Ga and Lfx exposure measured by LC–MS

**Figure 5:**
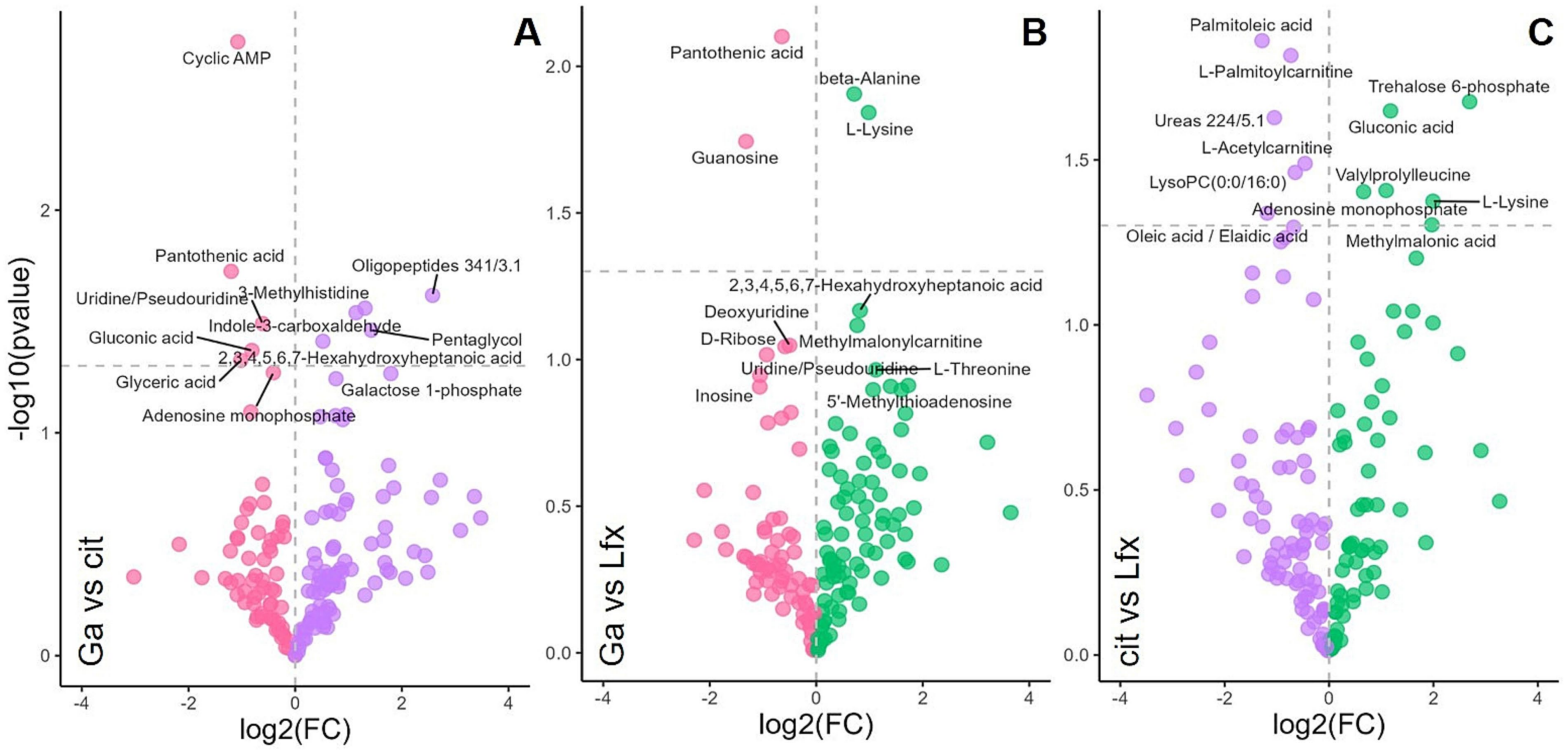
Effect of Ga and Lfx on the *Mtb* metabolome analyzed by LC–MS Volcano plot analysis identifies the most significant metabolites (marked with annotations) for every growth condition based on their *p*-value and fold change (FC); **(A)** metabolome of cells exposed to Ga compared to citrate control; **(B)** metabolome of cells exposed to Ga compared to Lfx; **(C)** metabolome of cells exposed to citrate compared to Lfx; pink – gallium; green – Ga/Lfx; purple – citrate.

GC–MS was the main approach used in this study for *Mtb* metabolome analysis. However, to increase the coverage of metabolites associated with Ga and Lfx exposure, we performed a focused LC–MS analysis on *Mtb* cells grown at 250 μM and 500 μM Ga as well as Lfx and citrate control (Figure 5). In the volcano plot analysis, the metabolites that significantly differed between citrate control and Lfx were distinct compared to those metabolites that significantly differed between Ga/Lfx and Ga (Figure 5; Supplementary Table 2). This supported the results from GC–MS that Ga altered *Mtb* metabolism in MGIT™ medium compared to citrate control and complemented the GC–MS data with several metabolites associated with Ga or Lfx exposure. Pantothenic acid, guanosine, cyclic-AMP, and uridine were significantly altered by Ga (Figure 5; Supplementary Table 2), while alanine and lysine levels were increased by Lfx when compared to Ga, which was in agreement with the increase of alanine in cells grown with Ga/Lfx measured by GC–MS (Figure 5; Supplementary Figure 5). Hence, Ga and Lfx may have an overlapping effect on the *Mtb* metabolome. However, some of the metabolites (and potentially metabolic pathways) may be affected distinctly by Ga and Lfx.

## Discussion

In this study, we observed that combinations of Ga citrate with established anti-TB antibiotics (Ga/Lfx and Ga/Lzd) have distinct effects on the growth and metabolite profile of drug-resistant *Mtb* strains. The effect differed depending on the concentration of Ga (250 or 500 μM). These are valuable findings for the potential future use of Ga in the treatment of drug-resistant TB (World Health Organization, 2023). The sequenced MDR-TB *Mtb* strain used for metabolomics belongs to the Beijing genotype (lineage 2.2.1), the most prevalent lineage in Asia and the WHO European Region (Merker et al., 2020). Hence, the study’s results may apply to this broad group within *Mtb* species and potentially other lineages.

Using metabolomics, we sought to understand the underlying mechanisms of Ga interaction with Lfx and Lzd. Ga interferes with the metabolism of iron in bacteria (Bernstein, 1998; Rodriguez et al., 2022). Different antibiotic classes, apart from their specific targets in the bacterial cell, can also interfere with bacterial metabolism (Lobritz et al., 2015). In our study, the concentrations of Lfx and Lzd were chosen to inhibit the growth of bacteria similarly. However, metabolite profiles of cells exposed to Lfx differed from those exposed to Lzd. Conversely, Ga/Lfx exhibited greater inhibitory effects on *Mtb* growth than Ga/Lzd. The metabolite profiles of cells grown in the presence of Ga/Lfx differed from those grown with Ga/Lzd.

Metabolome differences in cells grown with Ga/Lfx versus Ga/Lzd may be linked to or independent of the growth inhibition differences. For example, the stronger inhibiting effect of Ga/Lfx compared to Ga/Lzd, along with the changes in the metabolic profiles of the cells, may be linked to the differing mechanisms of action between Lfx and Lzd. Lfx is a bactericidal antibiotic that inhibits DNA gyrase and interferes with nucleic acid synthesis in bacteria (Mor et al., 1994), while Lzd inhibits protein synthesis and is a bacteriostatic antibiotic for Mtb (Myungsun et al., 2012). When Ga disables iron-containing enzymes, the expected response is a metabolism redirection toward less affected pathways. This redirection can occur through the transcription of genes associated with alternative pathways, which are less reliant on iron and, therefore, less sensitive to iron deficiency (Mor et al., 1994). However, such redirection of metabolism would be more difficult for cells grown in the presence of Lfx because of its inhibiting effect on transcription. A possible illustration of such decreased ability to redirect the metabolic pathways could be a Volcano plot comparison of Ga and Ga/Lfx at 500 μM (Figures 5A,B). It shows that glycine, sucrose, and a couple of unannotated metabolites are significantly upregulated in the presence of Ga and indicate a certain adaptation of metabolic pathways to sub-killing concentrations of Ga. However, in the presence of Ga/Lfx, only aconitic and fumaric acids and alanine levels increase (Figures 5A,B, Supplementary Figure 4). Taken together, this implies that at sub-killing concentrations of Ga, *Mtb* retains some capacity to redirect its metabolism to the routes less dependent on iron by producing new mRNA and synthesizing new enzymes.

On the other hand, the presence of Ga/Lfx resulted in fewer new upregulated metabolic products, implying that the bacteria may be unable to produce them and must rely only on the main household enzymes synthesized at the beginning of growth when iron was still available in the medium. While antibiotics at lethal concentrations are expected to strongly inhibit translation, which should theoretically prevent metabolic redirection, this study utilized antibiotic concentrations that inhibited only up to 10% of growth. Thus, Lzd should only have slowed down translation. Protein translation consumes up to 70% of cell energy (Stokes et al., 2019). When translation is decreased, cell metabolism is downregulated in response (Stokes et al., 2019; Biffo et al., 2024). Hence, a lower rate of metabolism due to translation inhibition may provide time and resources for *Mtb* to reorganize its metabolic pathways, avoiding those that depend on Fe. Similarly, during dormancy, *Mtb* lowers the metabolic flux of the cell, which decreases the killing potential of antibiotics (Stokes et al., 2019).

In general, as seen from the OPLS-DA models, the metabolite signature of Ga/Lfx exposure (increased levels of aconitic acid, fumaric acid, citric acid, alanine, and sugars) was overlapping with the metabolite signature of Ga exposure and, hence, we hypothesize that Ga and Lfx may, partly, affect the same metabolic pathways. Aconitic acid and fumaric acids are substrates of aconitase and fumarate hydratase, which are iron-containing enzymes involved in the TCA cycle enzymes that need iron for their activity, and aconitase was shown to be inhibited by Ga in previous studies (Olakanmi et al., 2013; Ruecker et al., 2017; Schwarz et al., 2021). Fluoroquinolones, including Lfx, can generate reactive oxygen species (ROS) (García et al., 2019), and ROS, in turn, similarly to Ga, inhibit enzymes containing iron–sulfur clusters and contribute to the killing of *Mtb* in the presence of fluoroquinolones (Shee et al., 2022). Even though the ROS generation is not the main mechanism of antibacterial activity for fluoroquinolones, it may enhance the Ga effect on *Mtb* central metabolism in the presence of Lfx. Alanine levels may increase due to alanine dehydrogenase activity that works in connection to the TCA cycle and catalyzes the reduction of pyruvate to alanine (Giffin et al., 2016).

Additionally, in this study, Ga led to the accumulation of such sugars as glucose, mannose, and galactose, indicating the inhibition of carbohydrate metabolism, which, in turn, is connected to DNA and RNA synthesis. Similarly, in another study, Ga inhibited *Mtb* ribonucleotide reductase (RR), an important iron-dependent enzyme for de-novo DNA synthesis connected to sugar metabolism through the pentose phosphate pathway (Olakanmi et al., 2013). Volcano plot analysis of LC–MS metabolomics data (Figures 5A,B) showed an accumulation of guanosine, uridine, and cyclic-AMP in the presence of Ga, which indicates alterations in nucleotide metabolism. In the case of the Ga/Lfx combination, any disturbances in nucleotide metabolism may compromise the synthesis and repair of nucleic acids and, in this way, strengthen the anti-nucleic acid activity of Lfx.

Interestingly, the metabolite profile of *Mtb* in the presence of Ga/Lfx, but not Ga/Lzd, was dependent on the concentration of Ga, as shown here by multivariate modeling (Figure 1C) and comparison of separate metabolites (Figure 3). This can either reflect the threshold concentration of Ga that can still be tolerated at the given level of transcription controlled by Lfx or may depend on the potential formation of the Ga–Lfx complex. Such complexes are not reported in the literature but are possible based on studies describing fluoroquinolone complexes with other metals. For example, Lfx can produce complexes with aluminum (Maeda et al., 2022), and enrofloxacin, which belongs to the fluoroquinolone class, can form a complex with iron (Sciscenko et al., 2021). In clinical practice, fluoroquinolones and ferrous sulfate are not recommended as they decrease the absorption of these antibiotics (Lehto et al., 1994). Our previous studies show that Ga complexes with different organic molecules can influence its uptake, antibacterial, anti-virulence, and anti-biofilm effects in bacteria (Rzhepishevska et al., 2011; Rzhepishevska et al., 2014).

The pH of the medium with citrate and Ga (Supplementary Figure 1) was lower than the standard pH of the Middlebrook 7H9 Broth used (Giffin et al., 2016). The advantages of using low pH in our study are the stability of Ga in the media and the similarity to pH that *Mtb* encounters in macrophages during infection (Gouzy et al., 2021; Early et al., 2019). However, the acidic pH of the growth medium can influence the metabolite profile and growth of *Mtb* (Gouzy et al., 2021). To address this, the experimental design included controls with the same acidity and citrate concentrations (Supplementary Figure 3).

The macrophage environment where *Mtb* resides during infection has a pH ranging from 6.2 to 4.5 (Early et al., 2019). According to Gouzy et al., *Mtb* grows well at pH as low as 4.5 in the presence of host lipids and their derivatives. Oleic acid is one of the substrates that secures *Mtb* growth at low pH (Gouzy et al., 2021). This agrees well with the findings in this study: *Mtb* provided with dextrose and oleic acid grew well even at pH 5 and started to show growth inhibition of about 50% only at pH lower than 4.5 (Supplementary Figure 1).

At pH below 5.5, *Mtb* starts switching to oleic acid as the preferred substrate, and the assimilation of oleic acid into metabolites of the citric acid cycle at pH 5 increases (Gouzy et al., 2021). In our study, the pH measured in Ga 500 uM samples and respective citrate controls was 5.7, which is higher than that used by Gouzy et al. However, it is likely that even in our study, *Mtb* at pH 5.7 switches to oleic acid consumption, at least partially, since *Mtb* usually uses several substrates simultaneously (de Carvalho et al., 2010). The switch to oleic acid should not block the TCA cycle activity because, similarly to dextrose, oleic acid is metabolized through the TCA cycle, entering it at the level of acetyl-CoA (Gouzy et al., 2021). In agreement with this, we did not see changes in the levels of TCA cycle acids apart from aconitate, citrate, and fumarate, which are connected to iron-containing enzymes. However, the metabolome as a whole can be influenced by the switch to a different carbon source. Hence, the difference between metabolomes at 250 uM and 500 uM Ga (Figure 3) can develop partially due to this switch. Taken together, the interaction of Ga with Lfx appears complex, may influence the biological activity of both agents, and is worth further study.

GC–MS was the main analytical approach in this study. However, we used LC–MS to look into the metabolite profile of Ga, Lfx, and citrate in more detail. This enabled us to add guanosine, cyclic-AMP, uridine, and pantothenic acid to the list of metabolites significantly increased in the presence of Ga (Figures 5A,B). In *Aspergillus fumigatus,* pantothenic acid synthesis was crucial for iron metabolism (Dietl et al., 2018). The involvement of pantothenic acid in iron metabolism in *Mtb* is still to be studied.

The MIC100 concentration of Ga in this study is quite high compared to other studies, being 1,000 μM, which corresponds to Ga 0.07 mg/L. This is because we used the standard Middlebrook 7H9 Broth that contains ferric ammonium citrate, 0.04 g/L (Ardito et al., 2001). This differs from most studies that use iron-free growth medium or *in vivo* testing, thereby decreasing the competition between iron and Ga and achieving Ga-related growth inhibition at lower concentrations (Olakanmi et al., 2013; Olakanmi et al., 2000). The *in vivo* concentration of Ga needed for the inhibitory effect is expected to be lower due to the lower competition with iron, which is largely sequestered by the host during the infection as one of the defense strategies (Bullen et al., 2005).

However, the same systems controlling iron (III) circulation will also sequester Ga ions. Therefore, predictions of which levels would be needed *in vivo* from *in vitro* experiments are challenging, and future pharmacokinetic studies in a relevant group are needed. Despite this, the results presented here suggest that Ga may be a good candidate for combination treatment of TB.

Based on the *in vitro* data presented in this study, we hypothesize that combining Lfx and Ga may improve treatment regimens *in vivo* and serve as an optional drug when other antibiotics cannot be used due to bacterial resistance or side effects. However, this hypothesis requires validation through *in vivo* testing, as the effects of iron sequestration and varying concentrations of Ga may greatly affect the outcome.

## Data Availability

Metabolomics data (raw cdf files) have been deposited to the EMBL-EBI MetaboLights database (Yurekten et al., 2024) with the identifier MTBLS11654, https://www.ebi.ac.uk/metabolights/MTBLS11654.

## Supplementary material

SUPPLEMENTARY FIGURE 1 *Mtb* growth inhibition in presence of Ga and citrate; **(A)** growth inhibition of *Mtb* MDR strain in MGIT^TM^ medium with different concentration of Ga (125-1000 μM) and citric acid (0.625 to 5mM) as control; **(B)** pH at different concentrations of Ga in MGIT™ medium.

SUPPLEMENTARY FIGURE 2 Loadings plot for PCA model summarizing all GC-MS metabolomics data; this loadings plot is to be used with the scores plot (Figure 1B) and shows the metabolites that are responsible for the separation of the samples in the model; together, both plots of the model show that samples (metabolomes of *Mtb* cell grown at different conditions) cluster according to two concentrations of Ga and/or citrate in the growth medium; model parameters: R2X [1] = 0.482; R2X [2] = 0.21; Ellipse: Hotelling's T2 (95%); coloured circles on the plot are used for visualisation and for matching the loadings plot groups to the scores plot.

SUPPLEMENTARY FIGURE 3 Influence of Ga on metabolome of *Mtb* compared to citrate control; **(A)** scores plot of a supervised OPLS-DA model; Scaled proportionally to R2X; R2X [1] = 0.452; R2Xo [1] = 0.273; Ellipse: Hotelling's T2 (95%); CV-ANOVA *p* =1.6e-06; the model shows sample separation based on experimental conditions: purple - citrate 1.25 and 2.5 mM; pink – Ga 250 and 500 μM; **(B)** the loadings plot of this model showing metabolites responsible for clustering; coloured circles on the plot are used for visualisation and for matching the loadings plot groups to the scores plot; the model indicates that the specific metabolite profile of gallium (metabolites highlighted in black/ bold in panel **B**) is independent of citrate exposure or pH; metabolites potentially responsible for pH response are highlighted in blue/ bold and arabinose (highlighted in purple/ bold in panel **B**) may be associated with citrate metabolism.

SUPPLEMENTARY FIGURE 4 Loadings plot for OPLS-DA model that illustrates *Mtb* metabolome differences between three experimental conditions at two concentrations of Ga; the loadings plot to be used with the scores plot of this model in Figure 1C; model parameters: scaled proportionally to R2X; R2X [1] = 0.289; R2X [2] = 0.241; Ellipse: Hotelling's T2 (95%); CV-ANOVA *p* =0.0001; coloured circles on the plot are used for visualisation and for matching the loadings plot groups to the scores plot; the model identifies the existence of two concentration-dependent subclusters in samples with Ga/Lfx.

SUPPLEMENTARY FIGURE 5 Peak area for alanine in cells exposed to Ga/Lfx 250 and 500 μM compared to other experimental conditions; **(A)** cells exposed to Ga/Lfx 250; **(B)** cells exposed to Ga/Lfx 500 μM; the levels of alanine in cells exposed to Ga/Lfx at different concentrations do not follow the pattern of Ga exposure presented in Figure 3; pink – Ga; green-blue – Ga/Lfx; green-yellow – Ga/Lzd.

SUPPLEMENTARY FIGURE 6 Comparison of Lfx and Lzd influence on *Mtb* metabolome of *Mtb*; **(A)** scores plot of a supervised OPLS-DA model; scaled proportionally to R2X; R2X [1] = 0.164; R2Xo [1] = 0.59; Ellipse: Hotelling's T2 (95%); CV-ANOVA *p* = 0,0005; the model shows sample separation based on experimental conditions: green - Lfx with citrate 1.25 and 2.5 mM; blue – Lzd with citrate 1.25 and 2.5 mM; **(B)** the loadings plot of this model showing metabolites responsible for clustering; coloured circles on the plot are used for visualisation and for matching the loadings plot groups to the scores plot.

SUPPLEMENTARY TABLE 1 Peak areas for all metabolites used in this study measured for all experimental conditions.

SUPPLEMENTARY TABLE 2 Summary of metabolites included in volcano plot analysis with *p* values and fold changes.

## References

[ref1] Ardito F. Posteraro B. Sanguinetti M. Zanetti S. Fadda G. (2001). Evaluation of BACTEC mycobacteria growth Indicator tube (MGIT 960) automated system for drug susceptibility testing of *Mycobacterium tuberculosis*. J. Clin. Microbiol. 39, 4440–4444. doi: 10.1128/JCM.39.12.4440-4444.2001, PMID: 11724858 PMC88562

[ref2] Belenky P. Ye J. D. Porter C. B. M. Cohen N. R. Lobritz M. A. Ferrante T.. (2015). Bactericidal antibiotics induce toxic metabolic perturbations that Lead to cellular damage. Cell Rep. 13, 968–980. doi: 10.1016/j.celrep.2015.09.059, PMID: 26565910 PMC4648786

[ref3] Bernstein L. R. (1998). Mechanisms of therapeutic activity for gallium. Pharmacol. Rev. 50, 665–682, PMID: 9860806

[ref4] Biffo S. Ruggero D. Santoro M. M. (2024). The crosstalk between metabolism and translation. Cell Metab. 36, 1945–1962. doi: 10.1016/j.cmet.2024.07.022 39232280 PMC11586076

[ref5] Bonchi C. Imperi F. Minandri F. Visca P. Frangipani E. (2014). Repurposing of gallium-based drugs for antibacterial therapy. Biofactors 40, 303–312. doi: 10.1002/biof.1159, PMID: 24532037

[ref6] Bullen J. J. Rogers H. J. Spalding P. B. Ward C. G. (2005). Iron and infection: the heart of the matter. FEMS Immunol. Med. Microbiol. 43, 325–330. doi: 10.1016/j.femsim.2004.11.010, PMID: 15708305

[ref7] CTV. (2024). IV Gallium study for patients with cystic fibrosis who have NTM (ABATE Study). Available at: (https://ctv.veeva.com/study/iv-gallium-study-for-patients-with-cystic-fibrosis-who-have-ntm-abate-study)

[ref8] de Carvalho L. P. S. Fischer S. M. Marrero J. Nathan C. Ehrt S. Rhee K. Y. (2010). Metabolomics of *Mycobacterium tuberculosis* reveals compartmentalized co-catabolism of carbon substrates. Chem. Biol. 17, 1122–1131. doi: 10.1016/j.chembiol.2010.08.009, PMID: 21035735

[ref9] Diacon A. H. Maritz J. S. Venter A. van Helden P. D. Andries K. McNeeley D. F.. (2010). Time to detection of the growth of *Mycobacterium tuberculosis* in MGIT 960 for determining the early bactericidal activity of antituberculosis agents. Eur. J. Clin. Microbiol. Infect. Dis. 29, 1561–1565. doi: 10.1007/s10096-010-1043-7, PMID: 20820832

[ref10] Dietl A. M. Meir Z. Shadkchan Y. Osherov N. Haas H. (2018). Riboflavin and pantothenic acid biosynthesis are crucial for iron homeostasis and virulence in the pathogenic mold *Aspergillus fumigatus*. Virulence 9, 1036–1049. doi: 10.1080/21505594.2018.1482181, PMID: 30052132 PMC6068542

[ref11] Early J. Ollinger J. Darby C. Alling T. Mullen S. Casey A.. (2019). Identification of compounds with pH-dependent bactericidal activity against *Mycobacterium tuberculosis*. ACS Infect. Dis. 5, 272–280. doi: 10.1021/acsinfecdis.8b00256, PMID: 30501173 PMC6371205

[ref12] García M. T. Valenzuela M. V. Ferrándiz M. J. de la Campa A. G. (2019). Reactive oxygen species production is a major factor directing the Postantibiotic effect of fluoroquinolones in *Streptococcus pneumoniae*. Antimicrob. Agents Chemother. 63, e00737–e00719. doi: 10.1128/AAC.00737-19 31160286 PMC6658797

[ref13] Giffin M. M. Shi L. Gennaro M. L. Sohaskey C. D. (2016). Role of alanine dehydrogenase of *Mycobacterium tuberculosis* during recovery from hypoxic nonreplicating persistence. PLoS One 11:e0155522. doi: 10.1371/journal.pone.0155522, PMID: 27203084 PMC4874671

[ref14] Goss C. (2022). A pharmacokinetic and safety study of IV Gallium Nitrate (Ganite) in cystic fibrosis patients. Available at: . (https://clinicaltrials.gov/ct2/show/NCT01093521)

[ref15] Gouzy A. Healy C. Black K. A. Rhee K. Y. Ehrt S. (2021). Growth of *Mycobacterium tuberculosis* at acidic pH depends on lipid assimilation and is accompanied by reduced GAPDH activity. Proc. Natl. Acad. Sci. USA 118:e2024571118. doi: 10.1073/pnas.2024571118, PMID: 34341117 PMC8364206

[ref16] Ilchenko A. (2021). Mevis. . (https://github.com/CreMoProduction/mevis).

[ref17] Knoll K. E. Lindeque Z. Adeniji A. A. Oosthuizen C. B. Lall N. Loots D. T. (2021). Elucidating the Antimycobacterial mechanism of action of ciprofloxacin using metabolomics. Microorganisms 9:1158. doi: 10.3390/microorganisms9061158, PMID: 34071153 PMC8228629

[ref18] Kontsevaya I. Lange C. Comella-Del-Barrio P. Coarfa C. DiNardo A. R. Gillespie S. H.. (2021). Perspectives for systems biology in the management of tuberculosis. Eur. Respir. Rev. 30:200377. doi: 10.1183/16000617.0377-2020, PMID: 34039674 PMC9488731

[ref19] Lehto P. Kivisto K. Neuvonen P. (1994). The effect of ferrous sulphate on the absorption of norfloxacin, ciprofloxacin and ofloxacin. Br. J. Clin. Pharmacol. 37, 82–85. doi: 10.1111/j.1365-2125.1994.tb04245.x, PMID: 8148225 PMC1364716

[ref20] Leitao R. C. F. Silva F. Ribeiro G. H. Santos I. C. Guerreiro J. F. Mendes F.. (2023). Gallium and indium complexes with isoniazid-derived ligands: interaction with biomolecules and biological activity against cancer cells and *Mycobacterium tuberculosis*. J. Inorg. Biochem. 240:112091. doi: 10.1016/j.jinorgbio.2022.112091, PMID: 36527994

[ref21] Liu S. F. Liu J. W. Lin M. C. Lee C. H. Huang H. H. Lai Y. F. (2007). Monitoring treatment responses in patients with pulmonary TB using serial lung gallium-67 scintigraphy. AJR Am. J. Roentgenol. 188, W403–W408. doi: 10.2214/AJR.06.0587, PMID: 17449733

[ref22] Lobritz M. A. Belenky P. Porter C. B. M. Gutierrez A. Yang J. H. Schwarz E. G.. (2015). Antibiotic efficacy is linked to bacterial cellular respiration. Proc. Natl. Acad. Sci. 112, 8173–8180. doi: 10.1073/pnas.1509743112, PMID: 26100898 PMC4500273

[ref23] Machado I. Marino L. B. Demoro B. Echeverría G. A. Piro O. E. Leite C. Q. F.. (2014). Bioactivity of pyridine-2-thiolato-1-oxide metal complexes: bi(III), Fe(III) and Ga(III) complexes as potent anti-*Mycobacterium tuberculosis* prospective agents. Eur. J. Med. Chem. 87, 267–273. doi: 10.1016/j.ejmech.2014.09.067 25261824

[ref24] Maeda Y. Takahashi Y. Naika Y. Maeda T. Otsuka Y. Saeki Y.. (2022). Ester prodrugs of levofloxacin to prevent chelate formation in presence of Aluminium ion. Pharm. Sci. 29, 65–74. doi: 10.34172/PS.2022.15

[ref25] Merker M. Nikolaevskaya E. Kohl T. A. Molina-Moya B. Pavlovska O. Brännberg P.. (2020). Multidrug- and extensively drug-resistant *Mycobacterium tuberculosis* Beijing clades, Ukraine, 2015. Emerg. Infect. Dis. 26, 481–490. doi: 10.3201/eid2603.190525, PMID: 32091369 PMC7045844

[ref26] Mor N. Vanderkolk J. Heifets L. (1994). Inhibitory and bactericidal activities of levofloxacin against *Mycobacterium tuberculosis* in vitro and in human macrophages. Antimicrob. Agents Chemother. 38, 1161–1164. doi: 10.1128/AAC.38.5.1161, PMID: 8067756 PMC188169

[ref27] Myungsun L. Jongseok L. Carroll M. W. Hongjo C. Seonyeong M. Taeksun S.. (2012). Linezolid for treatment of chronic extensively drug-resistant tuberculosis. N. Engl. J. Med. 367, 1508–1518. doi: 10.1056/NEJMoa1201964 23075177 PMC3814175

[ref28] Olakanmi O. Britigan B. E. Schlesinger L. S. (2000). Gallium disrupts iron metabolism of mycobacteria residing within human macrophages. Infect. Immun. 68, 5619–5627. doi: 10.1128/IAI.68.10.5619-5627.2000, PMID: 10992462 PMC101514

[ref29] Olakanmi O. Kesavalu B. Pasula R. Abdalla M. Y. Schlesinger L. S. Britigan B. E. (2013). Gallium nitrate is efficacious in murine models of tuberculosis and inhibits key bacterial Fe-dependent enzymes. Antimicrob. Agents Chemother. 57, 6074–6080. doi: 10.1128/AAC.01543-13, PMID: 24060870 PMC3837848

[ref30] Patel A. Graeff-Armas L. Ross M. Goldner W. (2020). “35–Hypercalcemia” in Abeloff’s clinical oncology. eds. Niederhuber J. E. Armitage J. O. Kastan M. B. Doroshow J. H. Tepper J. E. (Philadelphia: Elsevier).

[ref31] Patil V. Jain V. (2021). Understanding metabolic remodeling in *Mycobacterium smegmatis* to overcome energy exigency and reductive stress under energy-compromised state. Front. Microbiol. 12:722229. doi: 10.3389/fmicb.2021.722229 34539614 PMC8440910

[ref32] Reithuber E. Nannapaneni P. Rzhepishevska O. Lindgren A. E. G. Ilchenko O. Normark S.. (2020). The bactericidal fatty acid mimetic 2CCA-1 selectively targets pneumococcal extracellular polyunsaturated fatty acid metabolism. MBio 11, e03027–e03020. doi: 10.1128/mBio.03027-20 PMC7773995 33323510

[ref33] Riccardi N. Canetti D. Rodari P. Besozzi G. Saderi L. Dettori M.. (2020). Tuberculosis and pharmacological interactions: a narrative review. Curr. Res. Pharmacol. Drug Discov. 2:100007. doi: 10.1016/j.crphar.2020.100007 34909643 PMC8663953

[ref34] Rinschen M. M. Ivanisevic J. Giera M. Siuzdak G. (2019). Identification of bioactive metabolites using activity metabolomics. Nat. Rev. Mol. Cell Biol. 20, 353–367. doi: 10.1038/s41580-019-0108-4, PMID: 30814649 PMC6613555

[ref35] Rodriguez G. M. Sharma N. Biswas A. Sharma N. (2022). The Iron response of mycobacterium tuberculosis and its implications for tuberculosis pathogenesis and novel therapeutics. Front. Cell. Infect. Microbiol. 12:876667. doi: 10.3389/fcimb.2022.876667, PMID: 35646739 PMC9132128

[ref36] Ruecker N. Jansen R. Trujillo C. Puckett S. Jayachandran P. Piroli G. G.. (2017). Fumarase deficiency causes protein and metabolite succination and intoxicates *Mycobacterium tuberculosis*. Cell Chem. Biol. 24, 306–315. doi: 10.1016/j.chembiol.2017.01.005, PMID: 28219662 PMC5357164

[ref37] Rzhepishevska O. Ekstrand-Hammarström B. Popp M. Björn E. Bucht A. Sjöstedt A.. (2011). The antibacterial activity of Ga3+ is influenced by ligand complexation as well as the bacterial carbon source ▿. Antimicrob. Agents Chemother. 55, 5568–5580. doi: 10.1128/AAC.00386-11, PMID: 21947396 PMC3232821

[ref38] Rzhepishevska O. Hakobyan S. Ekstrand-Hammarström B. Nygren Y. Karlsson T. Bucht A.. (2014). The gallium(III)–salicylidene acylhydrazide complex shows synergistic anti-biofilm effect and inhibits toxin production by *Pseudomonas aeruginosa*. J. Inorg. Biochem. 138, 1–8. doi: 10.1016/j.jinorgbio.2014.04.009, PMID: 24837331

[ref39] Schwarz M. G. A. Antunes D. Corrêa P. R. da Silva-Gonçalves A. J. Malaga W. Caffarena E. R.. (2021). Mycobacterium tuberculosis and *M. bovis* BCG Moreau fumarate reductase operons produce different polypeptides that may be related to non-canonical functions. Front. Microbiol. 11:624121. doi: 10.3389/fmicb.2020.624121 33510737 PMC7835394

[ref40] Sciscenko I. Arques A. Varga Z. Bouchonnet S. Monfort O. Brigante M.. (2021). Significant role of iron on the fate and photodegradation of enrofloxacin. Chemosphere 270:129791. doi: 10.1016/j.chemosphere.2021.129791, PMID: 33556815

[ref41] Shee S. Singh S. Tripathi A. Thakur C. Kumar T. A. Das M.. (2022). Moxifloxacin-mediated killing of *Mycobacterium tuberculosis* involves respiratory downshift, reductive stress, and accumulation of reactive oxygen species. Antimicrob. Agents Chemother. 66, e00592–e00522. doi: 10.1128/aac.00592-22 35975988 PMC9487606

[ref42] Stokes J. M. Lopatkin A. J. Lobritz M. A. Collins J. J. (2019). Bacterial metabolism and antibiotic efficacy. Cell Metab. 30, 251–259. doi: 10.1016/j.cmet.2019.06.009, PMID: 31279676 PMC6990394

[ref43] Vanino E. Granozzi B. Akkerman O. W. Munoz-Torrico M. Palmieri F. Seaworth B.. (2023). Update of drug-resistant tuberculosis treatment guidelines: a turning point. Int. J. Infect. Dis. 130, S12–S15. doi: 10.1016/j.ijid.2023.03.013, PMID: 36918080

[ref44] Warrell R. P. Jr. Coonley C. J. Straus D. J. Young C. W. (1983). Treatment of patients with advanced malignant lymphoma using gallium nitrate administered as a seven-day continuous infusion. Cancer 51, 1982–1987. doi: 10.1002/1097-0142(19830601)51:11<1982::AID-CNCR2820511104>3.0.CO;2-L, PMID: 6839291

[ref45] Waxman A. D. Julien P. J. Brachman M. B. Tanasescu D. E. Ramanna L. Birnbaum F.. (1984). Gallium scintigraphy in bronchogenic carcinoma: the effect of tumor location on sensitivity and specificity. Chest 86, 178–183. doi: 10.1378/chest.86.2.178 6589118

[ref46] World Health Organization. (2023). Global tuberculosis report. Available at: (https://www.who.int/publications-detail-redirect/9789240083851).

[ref7001] Yurekten O. Payne T. Tejera N. Amaladoss F. X. Martin C. Williams M.. (2024). MetaboLights: open data repository for metabolomics. Nucleic Acids Research 52, D640–D646. doi: 10.1093/nar/gkad1045 37971328 PMC10767962

