# Supplementary figures and images for: Combination of gallium citrate and levofloxacin induces a distinct metabolome profile and enhances growth inhibition of multidrug-resistant Mycobacterium tuberculosis compared to linezolid
Source: Front Microbiol. 2024 Nov 29;15:1474071. doi: 10.3389/fmicb.2024.1474071 (PMC11654424; doi:10.3389/fmicb.2024.1474071)

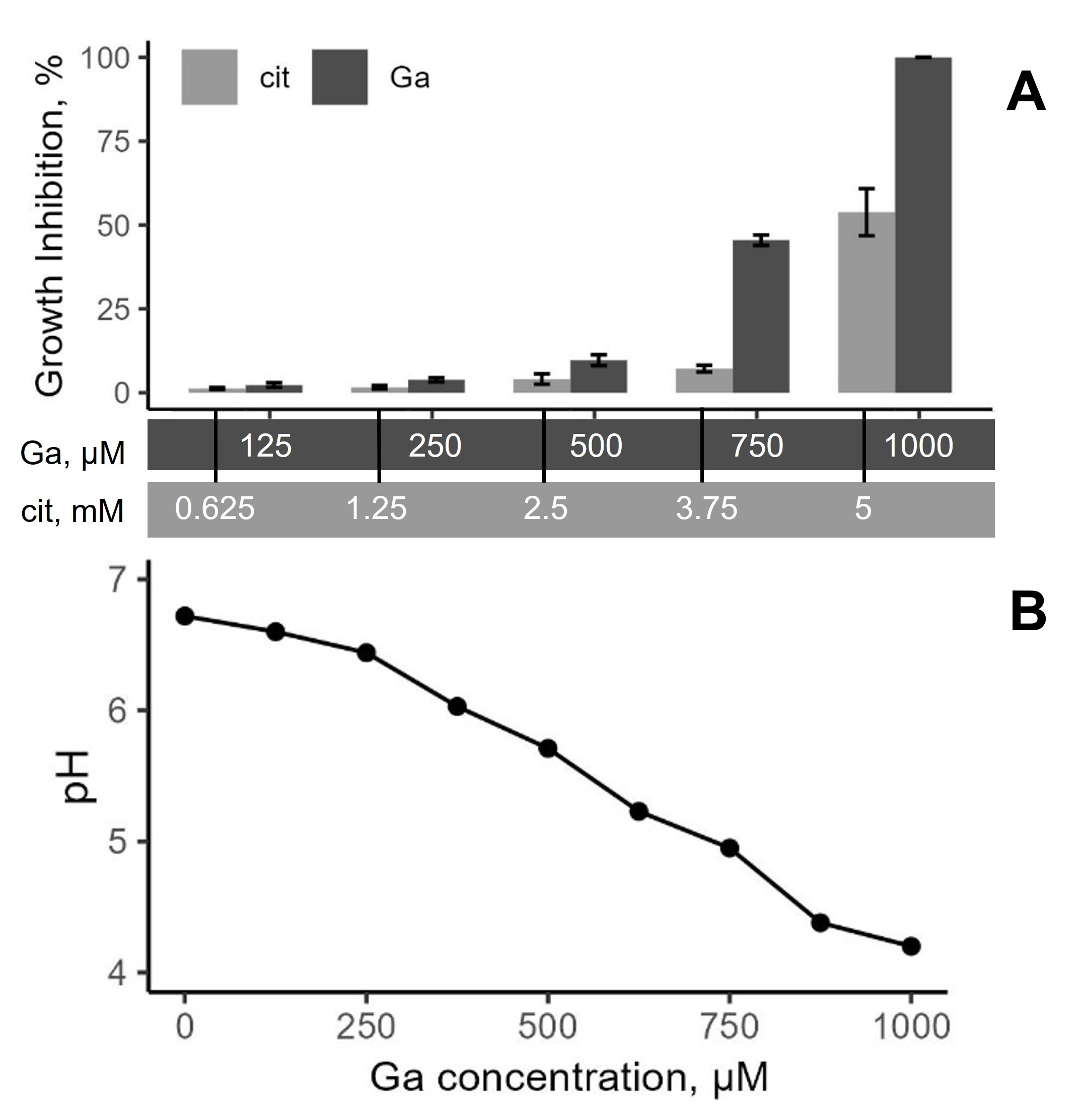

Supplement: SUPPLEMENTARY FIGURE 1 — Mtb growth inhibition in presence of Ga and citrate; (A) growth inhibition of Mtb MDR strain in MGITTM medium with different concentration of Ga (125-1000 μM) and citric acid (0.625 to 5mM) as control; (B) pH at different concentrations of Ga in MGIT™ medium. [file Image_1.jpeg]

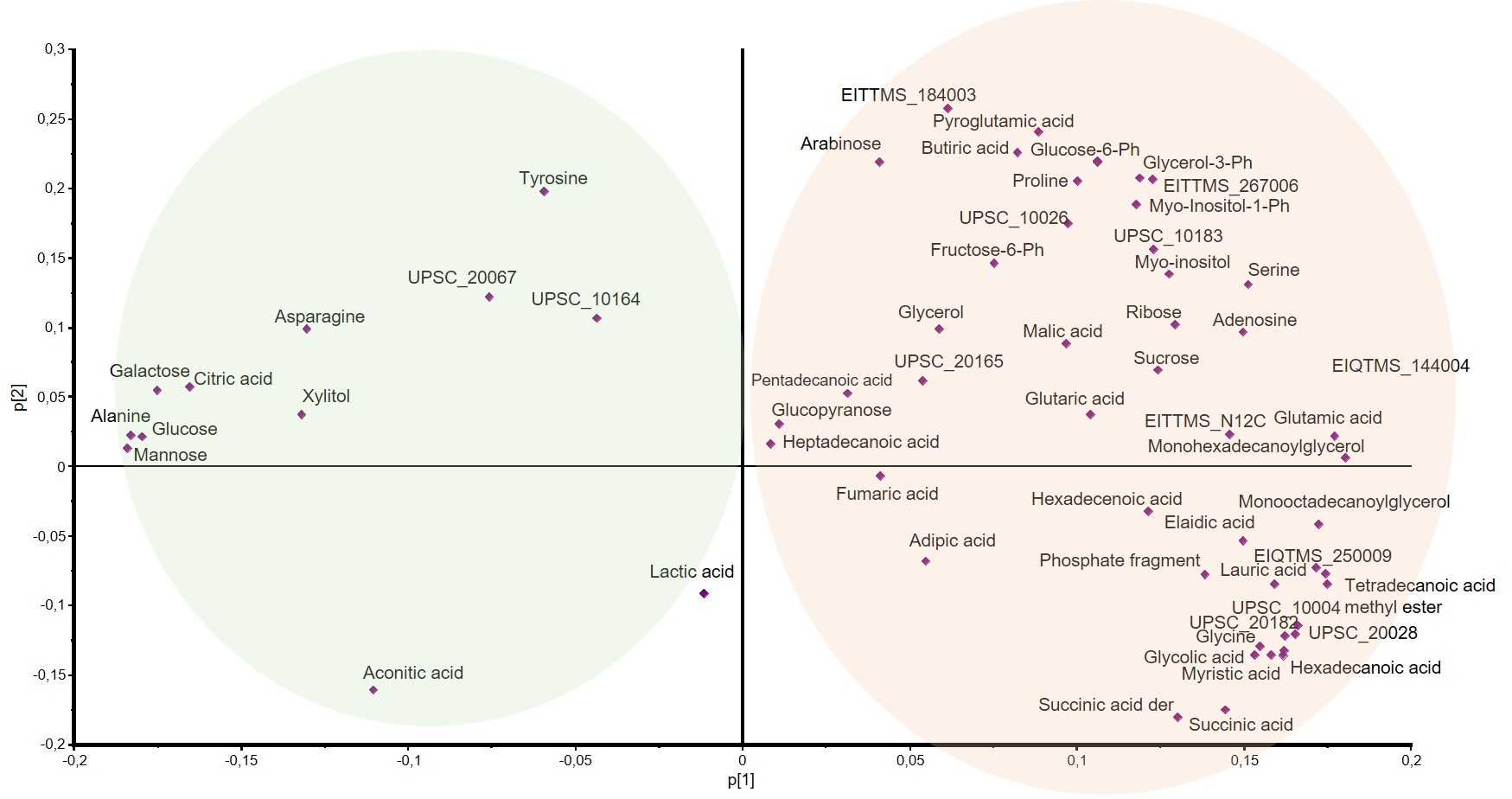

Supplement: SUPPLEMENTARY FIGURE 2 — Loadings plot for PCA model summarizing all GC-MS metabolomics data; this loadings plot is to be used with the scores plot (Figure 1B) and shows the metabolites that are responsible for the separation of the samples in the model; together, both plots of the model show that samples (metabolomes of Mtb cell grown at different conditions) cluster according to two concentrations of Ga and/or citrate in the growth medium; model parameters: R2X [1] = 0.482; R2X [2] = 0.21; Ellipse: Hotelling's T2 (95%); coloured circles on the plot are used for visualisation and for matching the loadings plot groups to the scores plot. [file Image_2.jpeg]

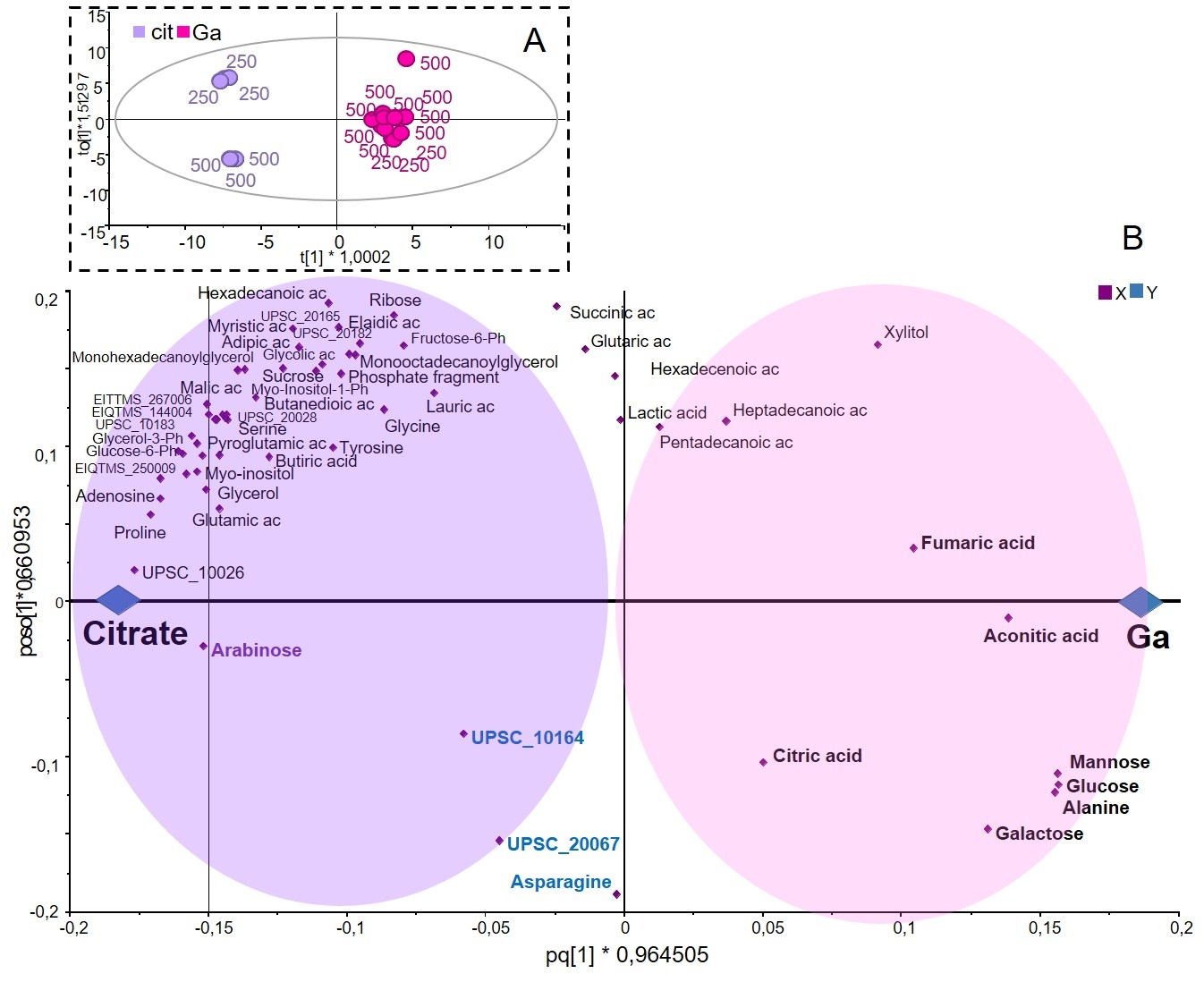

Supplement: SUPPLEMENTARY FIGURE 3 — Influence of Ga on metabolome of Mtb compared to citrate control; (A) scores plot of a supervised OPLS-DA model; Scaled proportionally to R2X; R2X [1] = 0.452; R2Xo [1] = 0.273; Ellipse: Hotelling's T2 (95%); CV-ANOVA p =1.6e-06; the model shows sample separation based on experimental conditions: purple - citrate 1.25 and 2.5 mM; pink – Ga 250 and 500 μM; (B) the loadings plot of this model showing metabolites responsible for clustering; coloured circles on the plot are used for visualisation and for matching the loadings plot groups to the scores plot; the model indicates that the specific metabolite profile of gallium (metabolites highlighted in black/ bold in panel B) is independent of citrate exposure or pH; metabolites potentially responsible for pH response are highlighted in blue/ bold and arabinose (highlighted in purple/ bold in panel B) may be associated with citrate metabolism. [file Image_3.jpeg]

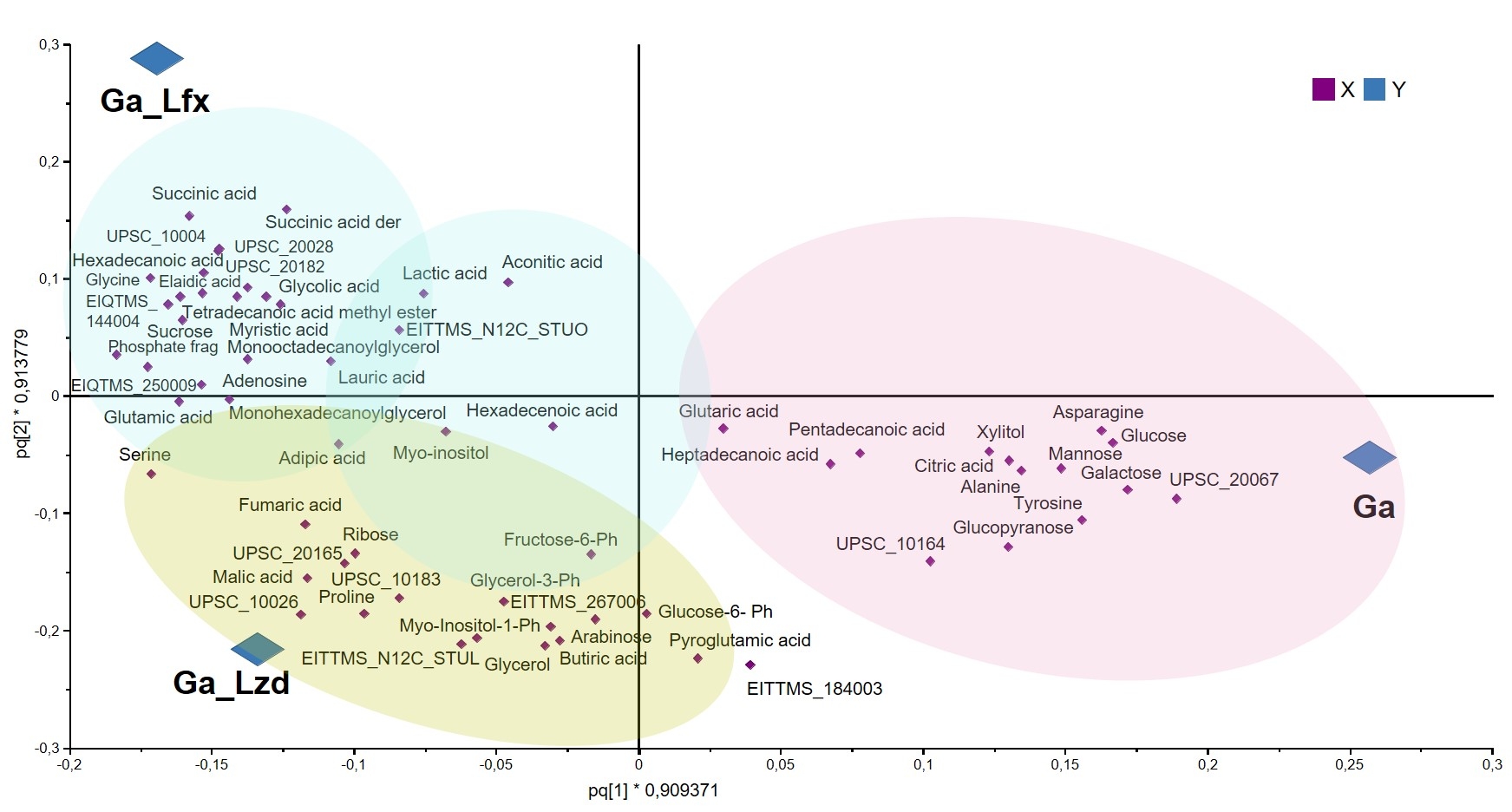

Supplement: SUPPLEMENTARY FIGURE 4 — Loadings plot for OPLS-DA model that illustrates Mtb metabolome differences between three experimental conditions at two concentrations of Ga; the loadings plot to be used with the scores plot of this model in Figure 1C; model parameters: scaled proportionally to R2X; R2X [1] = 0.289; R2X [2] = 0.241; Ellipse: Hotelling's T2 (95%); CV-ANOVA p =0.0001; coloured circles on the plot are used for visualisation and for matching the loadings plot groups to the scores plot; the model identifies the existence of two concentration-dependent subclusters in samples with Ga/Lfx. [file Image_4.jpeg]

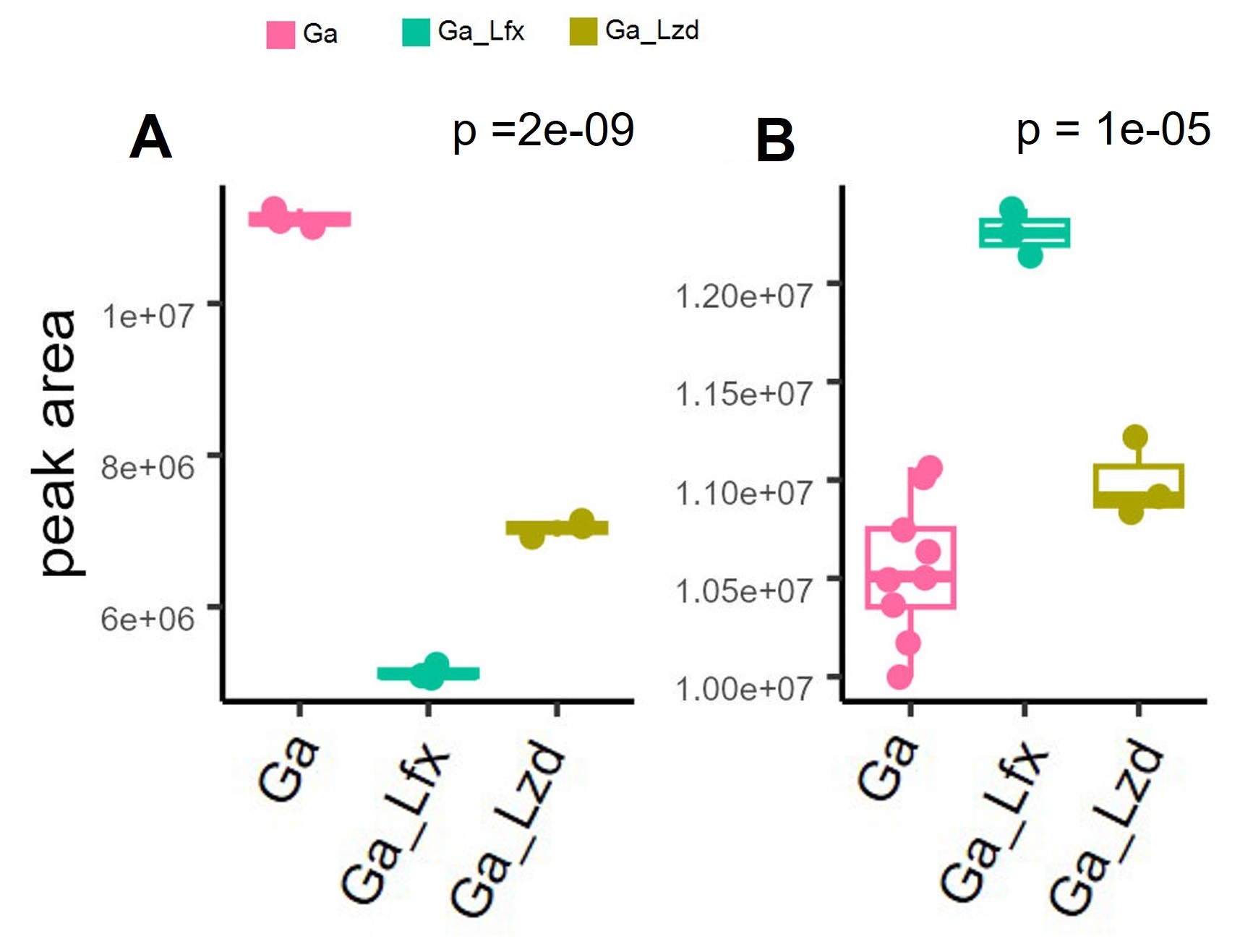

Supplement: SUPPLEMENTARY FIGURE 5 — Peak area for alanine in cells exposed to Ga/Lfx 250 and 500 μM compared to other experimental conditions; (A) cells exposed to Ga/Lfx 250; (B) cells exposed to Ga/Lfx 500 μM; the levels of alanine in cells exposed to Ga/Lfx at different concentrations do not follow the pattern of Ga exposure presented in Figure 3; pink – Ga; green-blue – Ga/Lfx; green-yellow – Ga/Lzd. [file Image_5.jpeg]

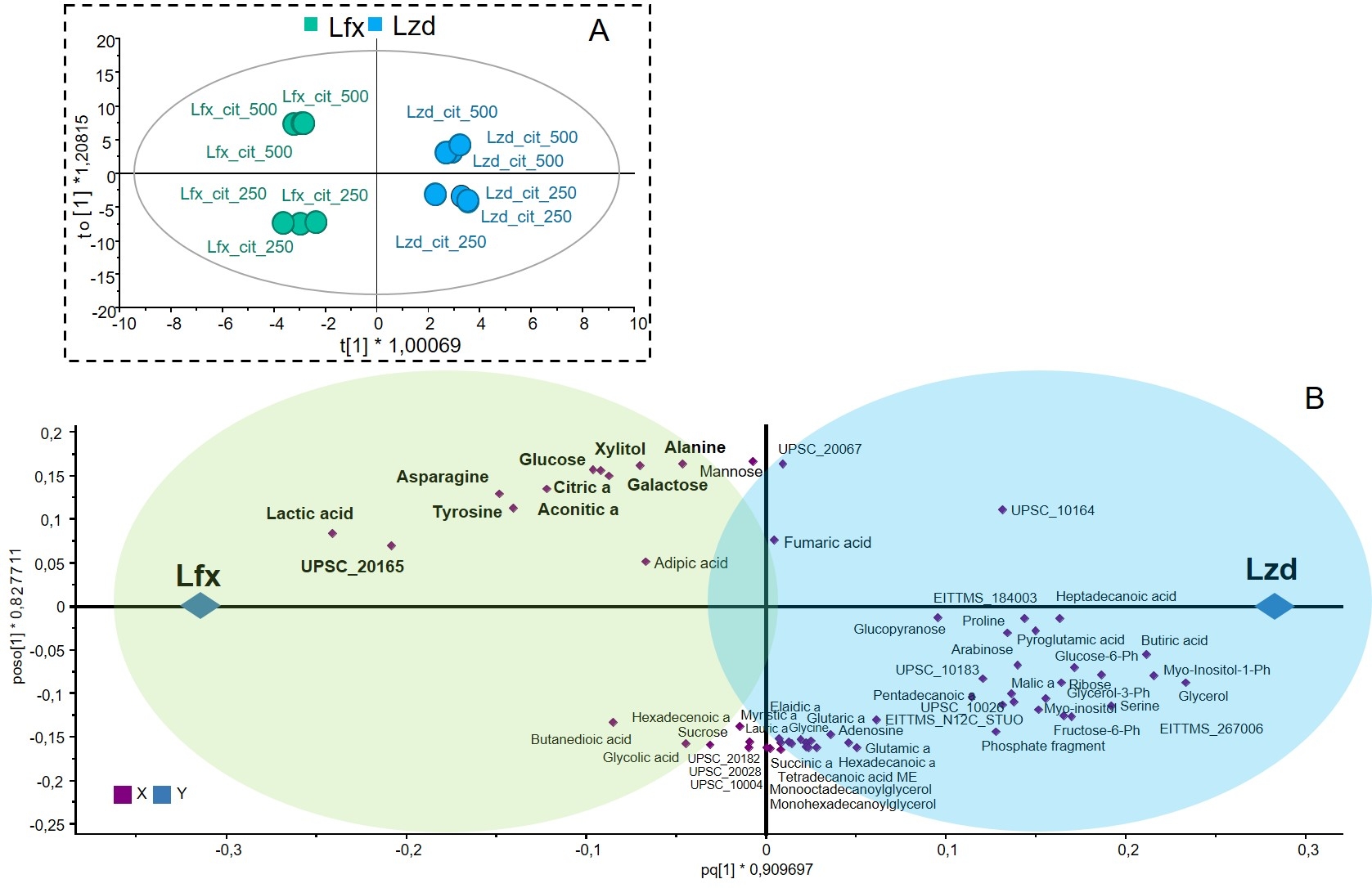

Supplement: SUPPLEMENTARY FIGURE 6 — Comparison of Lfx and Lzd influence on Mtb metabolome of Mtb; (A) scores plot of a supervised OPLS-DA model; scaled proportionally to R2X; R2X [1] = 0.164; R2Xo [1] = 0.59; Ellipse: Hotelling's T2 (95%); CV-ANOVA p = 0,0005; the model shows sample separation based on experimental conditions: green - Lfx with citrate 1.25 and 2.5 mM; blue – Lzd with citrate 1.25 and 2.5 mM; (B) the loadings plot of this model showing metabolites responsible for clustering; coloured circles on the plot are used for visualisation and for matching the loadings plot groups to the scores plot. [file Image_6.jpeg]
